# Design and synthesis of multi-functional small-molecule based inhibitors of amyloid-β aggregation: Molecular modeling and *in vitro* evaluation

**DOI:** 10.1371/journal.pone.0286195

**Published:** 2023-05-25

**Authors:** Awwad A. Radwan, Fars K. Alanazi, Mohammad Raish

**Affiliations:** 1 Kayyali Chair for Pharmaceutical Industries, Department of Pharmaceutics, College of Pharmacy, King Saud University, Riyadh, Saudi Arabia; 2 Department of Pharmaceutics, College of Pharmacy, King Saud University, Riyadh, Saudi Arabia; Universiti Teknologi Malaysia, MALAYSIA

## Abstract

Amyloid-β1–42 (Aβ42) peptide aggregate formation in the brain plays a crucial role in the onset and progression of Alzheimer’s disease. According to published research, the Aβ monomer’s amino acid residues KLVFF (16–20) self-associate to create antiparallel β-sheet fibrils. Small compounds can prevent self-assembly and destroy Aβ fibrils by attaching to the Aβ16–20 regions of Aβ42. To enhance biological characteristics and binding affinity to the amyloid beta peptide, β-sheet breaker small molecules can be developed and modified with various scaffolds. In the current study, a novel series of 2,3-disubstitutedbenzofuran derivatives was designed and created by fusing the benzofuran core of a known iron chelator, neuroprotective, and neurorestorative agent, like VK-28, with a motif found in the structure of a known muscarinic inhibitor and amyloid binding agent, like SKF-64346. Measurements of the binding affinity and in vitro aggregation inhibition of the Aβ42 peptide were made using the thioflavin T (ThT) test. Using AutoDock 4.2 software, molecular docking studies of the synthesized compounds were performed on the monomer and fibril structures of amyloid beta peptide. The compounds **8a–8g** exhibited strong binding energy and affinity to Aβ fibrils as well as a 50%–67% reduction of the growth of Aβ aggregation. Finally, the positive traits of our recently synthesized compounds make them excellent candidates for additional *in vivo* testing as a "β-sheet breaking agent."

## Introduction

**Fig 1:**
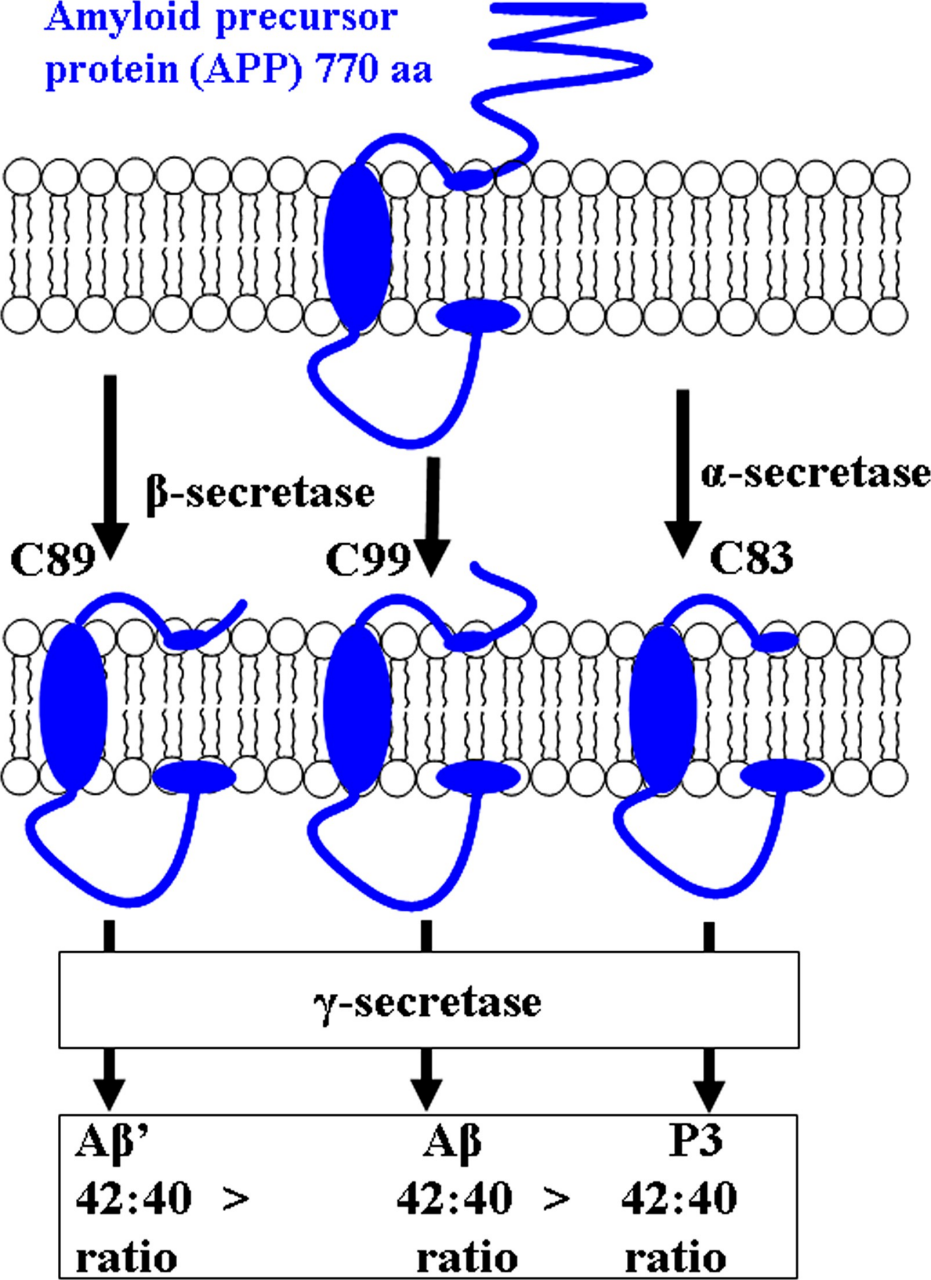
Generation of higher ratios of 42:40 for β-amyloid than for P3 peptides.

Alzheimer’s disease (AD) is a degenerative brain illness that impairs memory, reasoning, and reasoning skills, making daily tasks difficult. Since 1910, when the name AD was first coined, it has remained a hot topic and a challenging condition to understand for experts all around the world [1]. According to WHO figures, almost 55 million people worldwide suffer from dementia, with over 60% of them living in low- and middle-income countries. As the population of elderly people develops in almost every country, this number is expected to rise to 78 million in 2030 and 139 million in 2050 (https://www.who.int/news-room/fact-sheets/detail/dementia, accessed October 9, 2022). Over the last half-century, several advancements have been achieved in the study of neurodegenerative illnesses, and two main hypotheses have evolved to explain their progression. The first is known as "cholinergic," and it involves a decrease in acetylcholine (ACh) synthesis due to a decrease in choline acetyltransferase (ChAT) enzyme activity, a lack of transmitter axonal transport and choline reuptake, and a reduction in the number of nicotinic and muscarinic M2 central presynaptic receptors [2]. The second condition is known as "amyloid," and it involves an abnormal metabolism of the amyloid precursor protein (APP), a transmembrane glycoprotein that is typically controlled by sequential cleavage by beta-site APP-cleaving enzyme 1 (BACE1) and by the γ-secretase complex and resulting in short, soluble peptides. Normal Aβ40/42 production takes place in neuronal cells; the amount of produced peptides and the extent of the Aβ40/42 ratio are what distinguish pathological situations. In pathological conditions, the activities of β-secretase and γ-secretase produced two Aβ-peptides with lengths of 40 or 42 amino acids, which precipitate form fibrils via soluble oligomers, producing neuronal toxicity [3]. The so-called amyloidogenic pathway generates Aβ peptides by sequential proteolytic cleavage of the type-1 transmembrane amyloid precursor protein (APP) by two secretase entities, the aspartyl protease β-site APP cleaving enzyme 1 (BACE1, also known as β-secretase) and the tetrameric γ-secretase complex. β-Secretase can cleave APP at two different places in the APP sequence: Asp1 or Glu11, resulting in a 99 aa C-terminal fragment (C99) or an 89 aa CTF (C89) that is then processed by γ-secretase into full-length Aβ1-x (Aβ) or N-terminally truncated Aβ11-x (Aβ′) [4, 5]. While Aβ′ appears to be abundant species secreted by neurons and to contribute to the formation of insoluble aggregates in the brains of AD patients [6–8], Aβ1–42 and Aβ1–40 are the two most abundant Aβ species found in senile plaques. If APP is digested by α-secretase, which cleaves APP at the Aβ-17 location, resulting in 83 amino acids CTF (C83), which is then processed by α-secretase, releasing the p3 peptide (Aβ17-x), production of Aβ and Aβ′ is prevented. These 3 kDa peptides are not amyloidogenic, but they do play a role in the formation of non-congophilic, diffuse plaques in the cerebellum of Down syndrome patients and other brain regions in AD patients [9–11]. The carboxypeptidase-like trimming (γ-cleavage) of somewhat longer membrane bound peptides that are created during the initial endoproteolytic cleavage (ε- cleavage) of the individual amyloid precursor protein C-terminal fragments APP CTFs results in the different C-terminal variants of Aβ, Aβ′, and p3 that are produced by γ-secretase. The Aβ42: Aβ40 ratio is significantly different for all three peptide species (Aβ′ > Aβ > p3) (Fig 1) [12].

**Fig 2:**
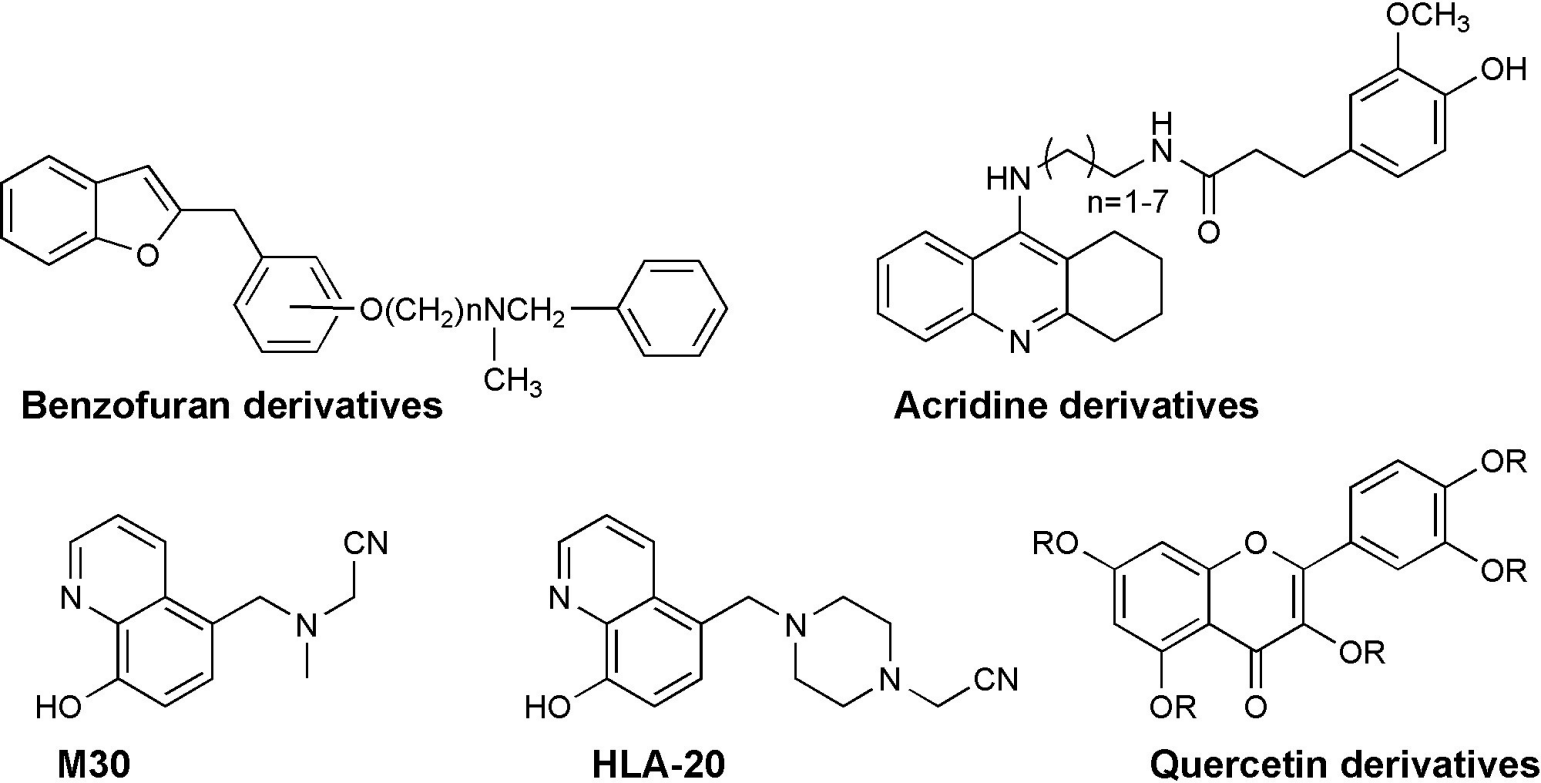
Representative compounds as multi-target anti-AD agents.

Other theories, such as intracellular deposition of hyper-phosphorylated tau protein neurofibrillary tangles, oxidative and inflammatory stress, all play a role in defining AD disease’s multifactor nature [13, 14]. Aβ aggregation and tau hyperphosphorylation are not separate processes but, rather are considered as closely interconnected. Recent research supports the idea that A causes tauopathy to expand into cortical regions, and that this spreading tauopathy is ultimately what causes cognitive impairment [15]. The traditional treatment for AD focuses on drugs that boost cholinergic neurotransmission by blocking acetylcholinesterase, such as rivastigmine, galantamine, or donepezil (E2020, Aricept®) [16]. While chemists and biologists have recently been interested in the role of butyryl cholinesterase inhibition in Alzheimer’s disease, butyl choline esterase has been discovered as a co-regulator of acetylcholine activity [17]. Furthermore, medication research strategies are currently focusing on preventing amyloid polymerization. Because AD is a complicated disease with multiple enzymes involved in its progression, changing only one protein may not be enough to produce the desired AD cure. In light of these findings, building multi-target compounds is a fascinating concept that could lead to a more effective therapeutic strategy [18]. Some researchers developed a series of hybrid chemicals based on this new paradigm, such as benzofuran and acridine derivatives (Fig 2). The benzofuran or acridine motif inhibits Aβ-aggregation in these hybrid drugs, while the alkylamine moiety inhibits cholinesterase (AChE and BuChE) [19–22]. A group of researchers revealed, in 1996, that acetylcholine esterase is prone to Aβ aggregation, with the peripheral anionic site playing a key role (PAS). Cell adhesion, neurite outgrowth, and amyloid deposition are all regulated by the PAS [23]. Several compounds (Fig 2) were designed to interact with both the central and PAS systems in order to have antiaggregant and anticholinesterase properties [24]. In addition, the existence of an iron-responsive region in the APP transcript’s 5’-UTR [25] revealed a strong relationship between iron metabolism and the AD pathogenic process. The development of iron-chelating neuroprotective neurorescue therapy is being combined with multi-modal tasks that act on several brain regions in recent emerging approaches. M30, HLA-20 (8-hydroxyquinoline derivatives) [26, 27], and quercetin analogues (polyphenol flavonoids) (Fig 2) showed the most action in terms of iron chelation, radical scavenging, and inhibiting iron-catalyzed lipid membrane peroxidation [28]. The ability of polyphenols to prevent Aβ aggregation has been attributed to the combined action of the phenolic moieties, which can either stack or interact hydrophobically with Aβ aromatic residues and insert into the space of Aβ aggregates, and/or interact via hydrogen bonding through phenolic hydroxyl groups [29].

**Fig 3:**
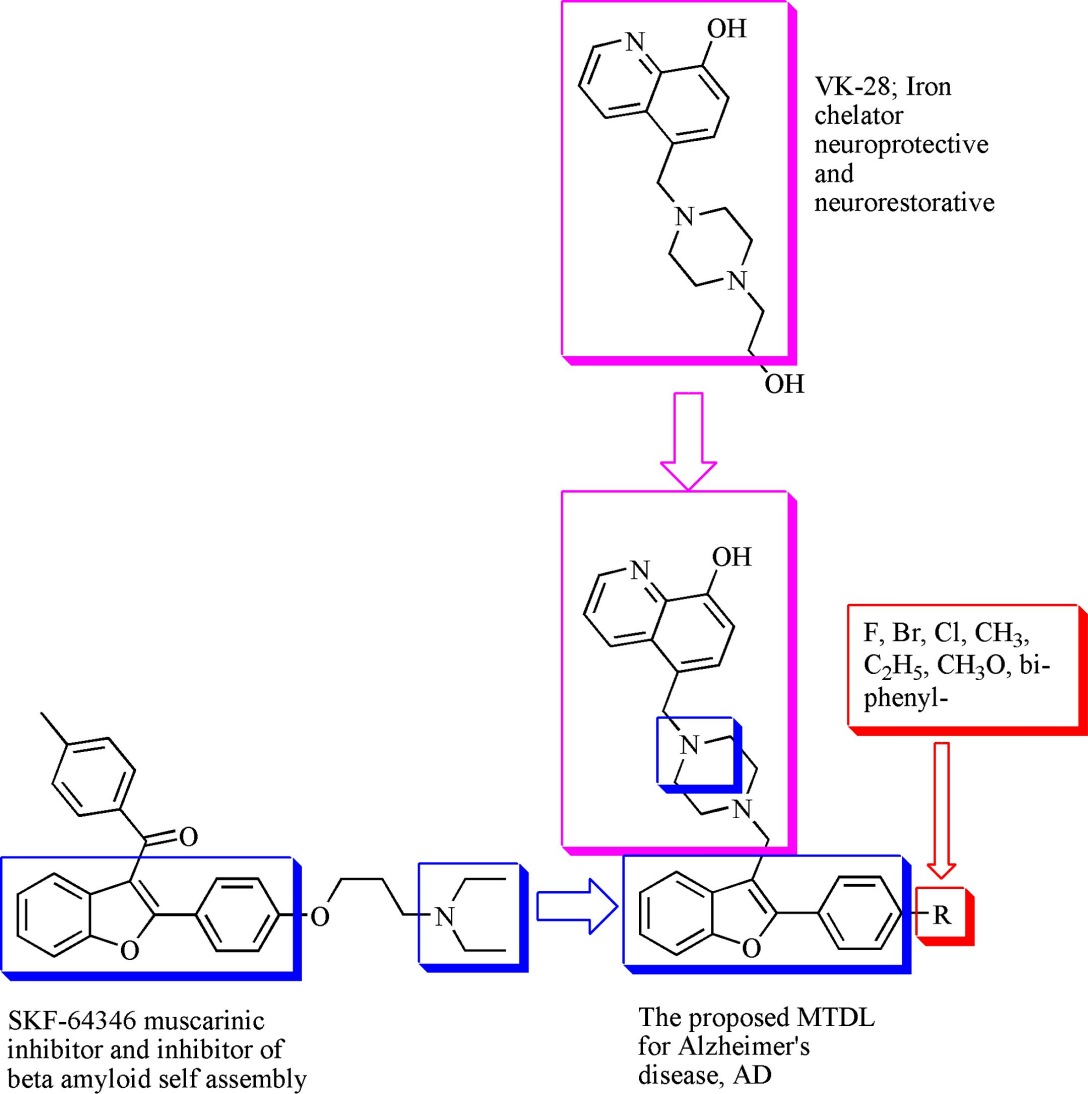
Schematic representation of the designed compounds.

In light of these findings, researchers considered developing a range of innovative multimodal drugs, such as AChE and Aβ-aggregation dual inhibitors clearly inspired by the SKF-46346 and structure and an iron-chelating neuroprotective neurorescue 8-hydroxyquinoline moiety. In our work we are prompted to synthesize a series of hybrid compounds (Fig 3) consisting of: a- benzofuran motif of SKF-64346, AChE/BChE inhibitors with inhibitor activity of Aβ-fibrill self aggregation. b- a substituted piperazine spacer, tertiary amine helps for cross BBB. c- *8-hydroxyquinoline* moiety, metal chelator, neuroprotective and neurorestorative. d- various substitutes on the aromatic ring at position 2 of benzofuran scaffold were included in order to study the substituent effect on the electronic configuration of the whole structure and its effect on the biological activity of the compounds.

## Experiment and procedures

### Chemistry

The starting materials and reagents were purchased from Sigma-Aldrich (St. Louis, MO, USA). Determination of the melting points was performed using an electrothermal melting point apparatus (Stuart Scientific, Stone, Staffordshire, UK), and were uncorrected. Precoated silica gel plates (Kieselgel 0.25 mm, 60G F254, Merck, Darmstadt, Germany) were used for thin layer chromatography (TLC) using chloroform/methanol (8:2) as developing system. All the chemical structure spectral analysis was done at the research center, College of Pharmacy, King Saud University, Saudi Arabia. Infrared (IR) spectra (KBr discs) were performed using a FTIR spectrophotometer (Perkin Elmer, Shelton, CT, USA). Nuclear magnetic resonance (NMR) spectra were obtained using NMR spectrophotometer (Bruker, Flawil, Switzerland) operating at for 1H and 125.76 MHz for 13C. Mass spectra were taken on a model 320 MS spectrometer (Varian, Lexington, KY, USA). Elemental analyses were performed on a model 2400 elemental analyzer (Perkin Elmer). Biological investigation experiment was done at the Stem Cell Therapy Program, King Faisal Specialized Hospital and Research Center.

#### Synthesis of 5-Chloromethyl-8-hydroxyquinoline hydrochloride 3

**Scheme 1:**
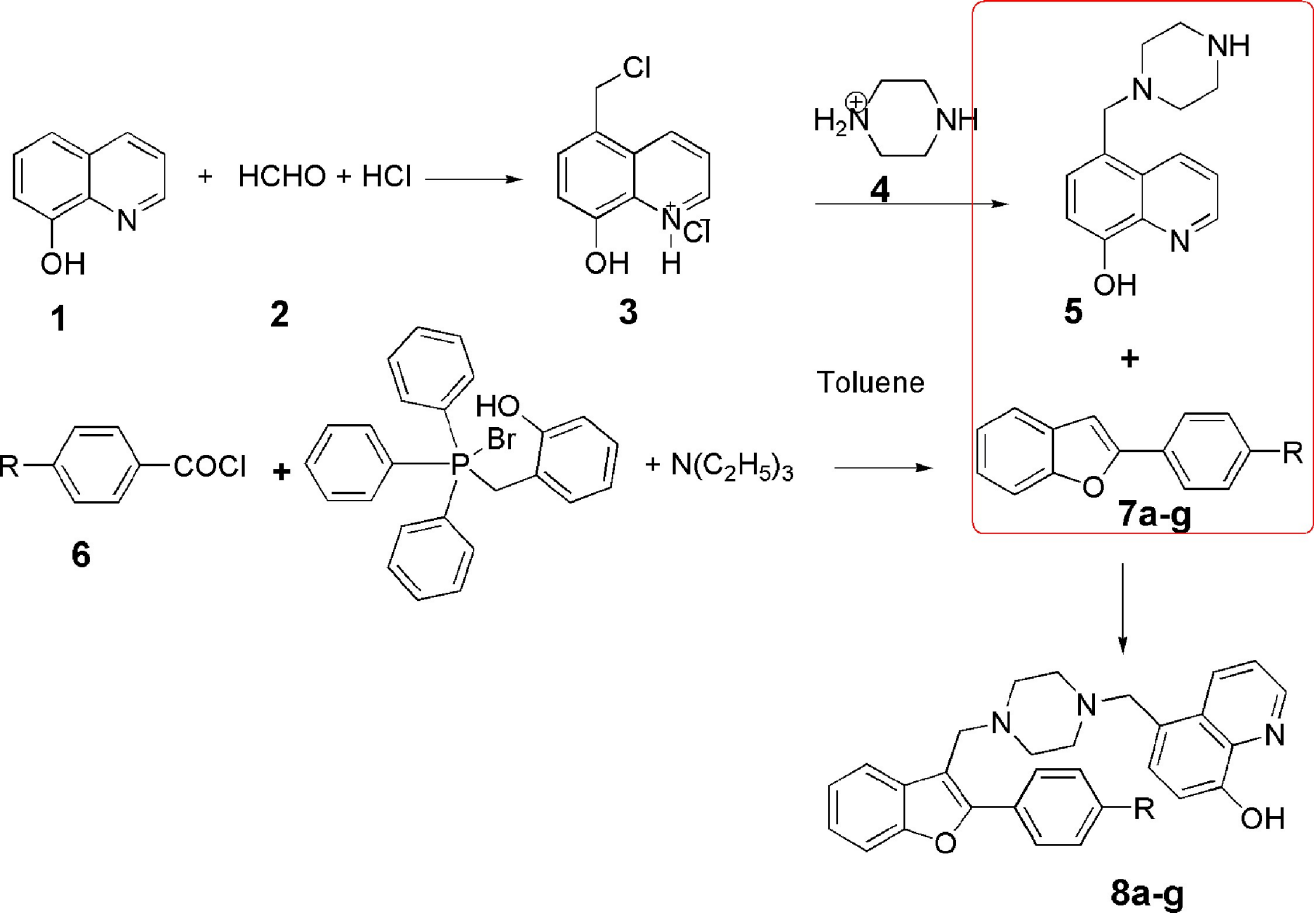
General synthesis of compounds 7a-g-8a-g. Reagents and conditions: (i) Et_3_N, toluene, reflux 10 hrs; (ii) Formaline 37%, DMF, stir 3 hr at r.t. R = C_2_H_5_, **a**; CH_3_O, **b**; F, **c**; Cl, **d**; Br, **e**; CH_3_, **f**; Biphenyl, **g.**

5-Chloromethyl-8-hydroxyquinoline hydrochloride (**3**) was synthesized through the reaction of conc HCl, 8-hydroxyquinoline (**1**) and formaline (**2**) (Scheme 1) according to the reported method, m.p. 282°C [30].

#### Synthesis of 5-((piperazin-1-yl)methyl)quinolin-8-ol 5

Piperazine anhydride (**4**, 1.9 g, 22 mmol) and piperazine dihydrochloride (6.36 g, 40 mmol) were dissolved in 20 ml of ethanol and then added to a three-necked flask equipped with a magnetic stir bar and reflux condenser. The flask was heated at 75°C for three hours while being vigorously stirred. Then, over the course of 30 minutes, 5-chloromethyl-8-hydroxyquinoline hydrochloride (**3,** 5.04 g, 22 mmol) solution was dropped gradually into the heated solution and the mixture was refluxed for additional 4 h. The reaction mixture was cooled, and the piperazine dihydrochloride that had precipitated was removed by filtration and washed with ethanol three times. The filtrate and washes were concentrated in vacuo and treated with saturated soda solution. The aqueous layer of crude resulting in 5-((piperazin-1-yl)methyl)quinolin-8-ol hydrochloride (**5**) was extracted into CH_2_Cl_2_ (3 × 15 ml). The combined organic extracts were dried over Na2SO4 anhydrous and were concentrated *in vacuo*. The obtained oily product was purified by flash column chromatography on silica gel (1:3 MeOH-CH_2_Cl_2_) to produce 5.0 g. (83%) of 5-((piperazin-1-yl)methyl)quinolin-8-ol (**5**).

Brown oil, yield 58%, ^1^H NMR (700 MHz, CDCl_3_): δ = 2.90 (4H, t, 2 CH_2_), 2.97 (4H, t, 2 CH_2_), 3.75 (2H, s, CH_2_), 7.20–8.53 (5H, m, ArH). ^13^CNMR (100 MHz, CDCl_3_): δ = 43.59, 48.69, 60.51, 115.52, 123.24, 128.38, 129.80, 132.74, 143.11, 144.2, 144.97, 150.15.

#### General synthesis of 2-(4-substitutedphenyl)benzofuran derivatives (7a-g)

A stirred suspension of 4-substitutedbenzoylchloride (5 g, 29 mmol), 2-hydroxybenzyltriphenyl phosphonium bromide (12 g, 27 mmol) and triethylamine (11.1 mL, 80 mmol) in toluene (125 mL) was refluxed for 10 hours. The reaction mixture was cooled and was concentrated under vacuum. Ethyl alcohol 150 mL were added to the oily residue and kept the refrigerator overnight. The obtained solid product is filtered off and dried.

#### 2-(4-Ethylphenyl)benzofuran (7a)

White solid, yield 92%, mp 100–101°C. IR (λmax, cm^-1^): 3042 (arom CH str), 2868 (aliph CH str), 805–736 (arom CH bend). ^1^H NMR (700 MHz, CDCl_3_): δ = 1.33 (3H, t, CH_3_), 2.75 (2H, q, CH_2_), 7.02–7.85 (9H, m, ArH). ^13^CNMR (100 MHz, CDCl_3_): δ = 15.51, 28.80, 100.64, 111.15, 120.79, 122.89, 124.03, 125.03, 128.03, 128.35, 129.41, 144.99, 154.84, 156.26. Ms m/z calcd: 221.1 [M]+, found: 222.0 [M]^+^ .Anal. calcd. for C_16_H_14_O: C, 86.45; H, 6.35. Found: C, 86.05; H, 6.52.

#### 2-(4-Methoxyphenyl)benzofuran (7b)

White solid, yield 92%, mp 150–151°C. IR (λmax, cm^-1^): 2956 (arom CH str), 2825 (aliph CH str), 799–741 (arom CH bend). ^1^H NMR (700 MHz, CDCl_3_): δ = 3.90 (3H, s, CH_3_O), 6.92–7.84 (9H, m, ArH). ^13^CNMR (100 MHz, CDCl_3_): δ = 55.39, 99.70, 111.01, 114.28, 120.60, 122.85, 123.37, 123.76, 126.44, 129.52, 154.72, 156.08, 160.01. Ms m/z calcd: 224.08 [M]^+^, found: 224.0 [M]^+^ .Anal. calcd. for C_15_H_12_O_2_: C, 80.34; H, 5.39. Found: C, 80.58; H, 5.61.

#### 2-(4-Fluorophenyl)benzofuran (7c)

White solid, yield 96%, mp 130–131°C. IR (λmax, cm^-1^): 3048 (arom CH str), 2832 (aliph CH str), 1213 (CF str), 837–741 (arom CH bend). ^1^H NMR (700 MHz, CDCl_3_): δ = 6.99–7.88 (9H, m, ArH). ^13^CNMR (100 MHz, CDCl_3_): δ = 101.03, 111.16, 115.85, 115.97, 120.92, 123.95, 124.32, 126.76, 126.81, 126.84, 129.21, 154.87, 162.02, 163.61. Ms m/z calcd: 212.06 [M]^+^, found: 212.0 [M]^+^ .Anal. calcd. for C_14_H_9_FO: C, 79.23; H, 4.27. Found: C, 79.02; H, 4.39.

#### 2-(4-Chlorophenyl)benzofuran (7d)

White solid, yield 90%, mp 110–111°C. IR (λmax, cm^-1^): 3130 (arom CH str), 826–742 (arom CH bend). ^1^H NMR (700 MHz, CDCl_3_): δ = 7.04–7.83 (9H, m, ArH). ^13^CNMR (100 MHz, CDCl_3_): δ = 101.77, 111.21, 116.18, 121.02, 123.11, 124.08, 125.76, 127.76, 128.88, 129.05, 131.23, 136.01, 153.78, 156.35. Ms m/z calcd: 228.03 [M]^+^, found: 227.98 [M]^+^ .Anal. calcd. for C_14_H_9_ClO C, 73.53; H, 3.97. Found: C, 73.33; H, 3.82.

#### 2-(4-Bromophenyl)benzofuran (7e)

Beige solid, yield 95%, mp 115–116°C. IR (λmax, cm^-1^): 3030 (arom CH str), 2835 (aliph CH str), 802–699 (arom CH bend). ^1^H NMR (700 MHz, CDCl_3_): δ = 7.1–7.98 (9H, m, ArH). ^13^CNMR (100 MHz, CDCl_3_): δ = 106.48, 111.45, 116.67, 119.34, 119.77, 121.04, 123.42, 124.01, 124.93, 126.55, 127.08, 127.2, 131.63, 149.02, 151.62. Ms m/z calcd: 271.98 [M]^+^, found: 271.67 [M]^+^ .Anal. calcd. for C_14_H_9_BrO C, 61.57; H, 3.32. Found: C, 61.36; H, 3.41.

#### 2-(*p*-Tolyl)benzofuran (7f)

White solid, yield 88%, mp 128–129°C. IR (λmax, cm^-1^): 3070 (arom CH str), 2906 (aliph CH str), 1492, 1436 (CH_3_ bend), 797–735 (arom CH bend). ^1^H NMR (700 MHz, CDCl_3_): δ = 2.44 (3H, s, CH_3_), 7.01–7.81 (9H, m, ArH). ^13^CNMR (100 MHz, CDCl_3_): δ = 21.29, 100.34, 111.12, 120.77, 124.03, 125.06, 127.61, 128.14, 129.07, 130.08, 138.45, 154.82, 156.15. Ms m/z calcd: 208.09 [M]^+^, found: 208.0 [M]^+^ .Anal. calcd. for C_15_H_12_O C, 86.51; H, 5.81. Found: C, 86.42; H, 5.88.

#### 2-Biphenylbenzofuran (7g)

White solid, yield 90%, mp 234–235°C. IR (λmax, cm^-1^): 3106 (arom CH str), 2854 (aliph CH str), 782–745 (arom CH bend). ^1^H NMR (700 MHz, CDCl_3_): δ = 7.2–7.98 (14H, m, ArH). ^13^CNMR (100 MHz, DMSO-d_6_): δ = 101.45, 111.20, 116.36, 120.93, 123.0, 125.17, 127.01, 128.27, 128.98, 129.05, 130.54, 132.02, 140.46, 142.42, 155.71, 157.18. Ms m/z calcd: 270.1 [M]^+^, found: 270.09 [M]^+^. Anal. calcd. for C_20_H_14_O C, 88.86; H, 5.22. Found: C, 88.68; H, 5.15.

#### General synthesis of 5-((4-((2-(4-substitutedphenyl)benzofuran-3-yl)methyl)piperazin-1-yl)methyl)quinolin-8-ol derivatives (8a-8g)

To a solution of 2-(4-Substitutedphenyl)benzofuran (7a-7g) (10 mmol) in DMF (10 mL), formaline (37%, 1.55 mL) and 5-((piperazin-1-yl)methyl)quinolin-8-ol (10 mmol) was added and stirred at r.t. for 3h. excess water have added and the mixture is kept in cold medium for 24 hrs. the prepared precipitate was filtered and washed with water, and then recrystallized from ethyl alcohol.5-((4-((2-(4-Ethylphenyl)benzofuran-3-yl)methyl)piperazin-1-yl)methyl)quinolin-8-ol (8a)

White solid, yield 85%, mp 130–131°C. IR (max, cm^-1^): 3353 (OH str), 3035 (arom CH str), 2929 (aliph CH str), 798–741 (arom CH bend). 1H NMR (700 MHz, CDCl_3_): δ = 1.21 (3H, t, CH_3_), 2.42 (4H, s, 2 CH_2_), 2.56 (2H, s, 2 CH_2_), 3.5 (2H, s, CH_2_), 3.51 (2H, s, CH_2_), 7.24–8.84 (13H, m, ArH). 13CNMR (100 MHz, CDCl_3_): δ = 20.36, 33.40, 45.35, 55.21, 55.99, 57.48, 111.50, 115.75, 115.86, 121.91, 120.91, 125.62, 125.74, 126.15, 127.83, 128.96, 129.63, 129.73, 132.47, 133.21, 134.00, 137.80, 138.48, 149.77, 159.42. Ms m/z calcd: 477.24 [M]^+^, found: 477.09 [M]+ .Anal. calcd. for C_31_H_31_N_3_O_2_: C, 77.96; H, 6.54; N, 8.80. Found: C, 78.02; H, 6.37; N, 8.91.

#### 5-((4-((2-(4-Methoxyphenyl)benzofuran-3-yl)methyl)piperazin-1-yl)methyl)quinolin-8-ol (8b)

White solid, yield 80%, mp 141–142°C. IR (λmax, cm^-1^): 3345 (OH str), 3058 (arom CH str), 2949 (aliph CH str), 799–744 (arom CH bend). 1H NMR (700 MHz, DMSO-d_6_): δ = 2.73 (4H, s, 2CH_2_), 2.89 (4H, s, 2CH_2_), 3.41 (4H, s, 2CH_2_), 3.82 (3H, s, CH_3_O), 7.06–8.85 (13H, m, ArH). 13CNMR (100 MHz, DMSO-d_6_): δ = 40.86, 52.05, 52.69, 55.55, 59.12, 111.14, 112.72, 114.28, 116.48, 120.60, 121.44, 122.65, 123.11, 123.65, 124.51, 126.12, 126.88, 127.84, 128.48, 131.52, 136.34, 148.14, 150.40, 151.03, 152.48, 159.18. Ms m/z calcd: 479.22 [M]^+^, found: 479.75 [M]^+^ .Anal. calcd. for C_30_H_29_N_3_O_3_ C, 75.13; H, 6.10; N, 8.76. Found: C, 74.95; H, 6.24; N, 8.62.

#### 5-((4-((2-(4-Fluorophenyl)benzofuran-3-yl)methyl)piperazin-1-yl)methyl)quinolin-8-ol (8c)

White solid, yield 75%, mp 118–119°C. IR (λmax, cm^-1^): 3328 (OH str, 3052 (arom CH str), 2923 (aliph CH str), 837–743 (arom CH bend). 1H NMR (700 MHz, DMSO-d_6_): δ = 2.50 (4H, m, 2CH_2_), 2.54 (4H, m, 2CH_2_), 3.60 (5H, brs, 2CH_2_, OH), 3.69 (2H, s, 2CH_2_), 7.24–8.98 (13H, m, ArH). 13CNMR (100 MHz, DMSO-d_6_): δ = 40.38, 52.26, 52.79, 58.16, 111.42, 112.67, 115.94, 117.19, 120.85, 121.33, 123.61, 124.45, 124.73, 126.04, 126.39, 126.77, 128.15, 129.01, 132.01, 137.21, 150.15, 151.18, 151.72, 154.14, 163.12. Ms m/z calcd: 467.20 [M]^+^, found: 467.83 [M]^+^. Anal. calcd. for C_29_H_26_FN_3_O_2_ C, 74.50; H, 5.61; N, 8.99. Found: C, 74.35; H, 5.52; N, 8.81.

#### 5-((4-((2-(4-Chlorophenyl)benzofuran-3-yl)methyl)piperazin-1-yl)methyl)quinolin-8-ol (8d)

White solid, yield 90%, mp 134–135°C. IR (λmax, cm^-1^): 3358 (OH str), 3058 (arom CH str), 2927 (aliph CH str), 823–745 (arom CH bend). 1H NMR (700 MHz, CDCl_3_): δ = 2.73 (4H, s, 2 CH_2_), 2.89 (4H, s, 2 CH_2_), 3.37 (2H, s, CH_2_), 3.71 (2H, s, CH_2_), 6.97–8.84 (13H, m, ArH). 13CNMR (100 MHz, CDCl_3_): δ = 41.20, 52.26, 56.83, 64.74, 110.76, 115.96, 116.27, 120.86, 125.85, 127.97, 128.91, 129.45, 130.60, 130.98, 132.48, 132.67, 133.54, 133.60, 133.65, 133.75, 133.80, 134.20, 136.00, 152.98, 158.44. Ms m/z calcd: 483.17 [M]+, found: 483.65 [M]+ .Anal. calcd. for C_29_H_26_ClN_3_O_2_ C, 71.97; H, 5.41; N, 8.68. Found: C, 72.12; H, 5.35; N, 8.54.

#### 5-((4-((2-(4-Bromophenyl)benzofuran-3-yl)methyl)piperazin-1-yl)methyl)quinolin-8-ol (8e)

Beige solid, yield 70%, mp 115–116°C. IR (λmax, cm^-1^): 3361 (OH str, 3055 (arom CH str), 2928 (aliph CH str), 819–709 (arom CH bend). 1H NMR (700 MHz, CDCl_3_): δ = 2.73 (4H, s, 2 CH_2_), 2.89 (4H, s, 2 CH_2_), 3.41 (2H, s, CH_2_), 3.82 (2H, s, CH_2_), 7.06–8.5 (13H, m, ArH). ^13^CNMR (100 MHz, CDCl_3_): δ = 41.12, 52.05, 52.63, 58.29, 112.92, 115.90, 116.22, 120.94, 126.01, 127.17, 128.04, 128.97, 129.53, 130.75, 131.26, 132.69, 132.94, 133.35, 133.75, 134.01, 134.61, 136.11, 136.81, 141.13, 158.49. Ms m/z calcd: 527.12 [M]^+^, found: 527.61 [M]^+^. Anal. calcd. for C_29_H_26_BrN_3_O_2_ C, 65.91; H, 4.96; N, 7.95. Found: C, 65.78; H, 4.82; N, 8.08.

#### 5-((4-((2-p-tolylbenzofuran-3-yl)methyl)piperazin-1-yl)methyl)quinolin-8-ol (8f)

White solid, yield 78%, mp 116–117°C. IR (λmax, cm-1): 3361 (OH str), 2933 (arom CH), 3045 (arom CH str), 1452, 1370 (CH_3_ bend), 2933 (aliph CH str), 798–738 (arom CH bend). 1H NMR (700 MHz, DMSO-d_6_): δ = 2.32 (3H, s, CH_3_), 2.77 (4H, s, 2 CH_2_), 2.98 (2H, s, 2CH_2_), 3.39 (5H, br s, two CH_2_, OH), 6.96–7.90 (13H, m, ArH). 13CNMR (100 MHz, DMSO-d_6_): δ = 26.13, 36.11, 41.24, 48.20, 50.23, 111.48, 115.82, 125.59, 125.70, 127.79, 128.92, 129.51, 129.62, 130.87, 132.23, 133.98, 134.35, 137.60, 143.42, 159.31, 160.6. Ms m/z calcd: 463.23 [M]^+^, found: 463.62 [M]^+^. Anal. calcd. for C_30_H_29_N_3_O_2_ C, 77.73; H, 6.31; N, 9.06. Found: C, 77.61; H, 6.25; N, 9.02.

#### 5-((4-((2-Biphenylbenzofuran-3-yl)methyl)piperazin-1-yl)methyl)quinolin-8-ol (8g)

White solid, yield 65%, mp 206–207°C. IR (λmax, cm^-1^): 3344 (OH str), 3085 (arom CH str), 2912 (aliph CH str), 794–692 (arom CH bend). 1H NMR (700 MHz, CDCl_3_): δ = 2.19 (8H, s, 4CH_2_), 2.90 (2H, s, CH_2_), 2.96 (2H, s, CH_2_), 6.93–8.21 (18H, m, ArH). 13CNMR (100 MHz, DMSO-d_6_): δ = 40.59, 51.38, 52.11, 57.65, 111.34, 112.38, 116.89, 120.93, 121.18, 123.0, 123.48, 125.05, 125.91, 126.5, 127.12, 127.52, 128.22, 128.36, 129.05, 130.13, 132.05, 136.01, 136.85, 136.72, 148.46, 150.41, 151.23, 154.12. Ms m/z calcd: 525.24 [M]^+^, found: 525.63 [M]^+^. Anal. calcd. for C_35_H_31_N_3_O_2_ C, 79.97; H, 5.94; N, 7.99. Found: C, 79.82; H, 5.85; N, 7.91.

### Thioflavin T (ThT) assay to study Aβ aggregation

The SensoLyte® Thioflavin T ß-Amyloid42 Aggregation Kit was purchased from AnaSpec Inc. (34801 Campus Drive Fremont CA 94555 USA). To prevent self-aggregation, hexafluoroisopropanol (HFIP) at a concentration of 5 mg/ml was first applied to Aβ1–42 peptides. The peptide-containing clear solution was then aliquoted into a micro centrifuge tube. Under a stream of nitrogen, the HFIP was evaporated until a transparent film was all that was left in the test tube. After that, DMSO was used to dissolve the pre-treated Aβ1–42 samples to create a stable stock solution (Aβ1–42 1 mM). The tested substance (25 mM) was either present or absent in a combination containing the peptide (10 mL, 50 mM, final concentration) that was incubated at 37°C for 48 hours. Using the thioflavin-T fluorescence technique, the production of amyloid fibrils was quantified. Additionally, blank tests using Aβ1–42 with or without inhibitors and 50 mM phosphate buffer (pH 7.4) were run. Following incubation, samples were diluted with 50 mM glycine NaOH buffer (pH 8.0) containing thioflavin-T to create a final volume of 200 mL. (5 mM). Five minutes later, the fluorescence intensities were measured (excitation, 440 nm; emission, 484 nm). The expression (1-IFi/IFc) x 100% was used to compute the percentage of inhibition of aggregation, where IFi and IFc are the fluorescence intensities obtained for Ab in the presence and absence of inhibitors, respectively, after removing background. Three copies of each measurement were made [31, 32].

### Determination of ligand/iron(III) stoichoimetries using UV/Vis spectrophotometry

Analytical reagent grade ferric chloride was dissolved in 100% ethanol to create a stock solution of ferric chloride (1.35 X 10–4 M). Compound 8e was dissolved in ethanol to create the ligand solution (1.35 X 10–4 M). On a Shimadzu UV-2550 spectrophotometer, UV spectra were captured. The stoichiometric ratios of the iron(III) model systems with compound 8e were calculated using a spectrophotometric technique described by Joe [33]. For a range of iron(III) and ligand mixes, the amount of iron(III)-ligand complex was determined while maintaining a constant volume of 10.0 mL for the entire mixture. The complex’s stoichiometric composition was established in light of the results.

### ADMET prediction

Using the ChemDraw program (ChemDraw® Ultra, version 12, 1986–2009, Cambridge Soft Corp., USA), the structures of compounds 8a–g were shown. The import icon in the molecular sketcher was clicked, opening a new window where the produced structure could be selected and subsequently transferred to the SMILES format on the SwissADME server, http://www.swissadme.ch (accessed on 15 April 2020). The "Run" icon was then clicked, supplying the ADMET settings and associated values.

### Molecular docking

The 3D model of compounds **8a - 8g** with the lowest energy levels was chosen for molecular docking studies. The protein data base PDB was initially used to obtain the crystal structures of the human Aβ amyloid monomer (Aβ m) [34] and Aβ amyloid fibril (Aβ f) [35]. The Lamarckian genetic approach molecular docking was performed using AutoDock 4.2 software [36]. The polar hydrogen atoms and Kollman charges of the ligands and Aβ1–42 monomer were identified after the nonpolar hydrogen atoms were mixed using AutoDock tools and saved in.pdbqt format. The ligands were kept flexible and the Aβ1–42 monomer was kept rigid in the grid box during the docking method to establish the ligands’ free rotation around it. An AutoGrid grid box covering the entire Aβ1–42 monomer structure was made with dimensions of 126 X 68 X 68 Å, grid spacing 0.375 Å, and coordinate centers X = -1.739, Y = 1.658, and Z = -6.771 in order to compare the binding energies between the synthesized compounds and the Aβ1–42 monomer and to determine the best structural conformation. Ten docked conformations that all achieved structures with a tolerance of two were constructed for root mean square deviation (RMSD), and their docking energies were sorted to provide the findings. 

## Results and discussions

### Chemistry

Scheme 1 shows the synthesis pathway of compounds –**7a,b-8a,b**. Compounds 2-(4-substitutedphenyl)benzofuran derivatives (**7a,b**) were obtained through the Wittig reaction that is an easy procedure for the synthesis of 3-Aroyl[b]benzofurans through the reaction of aroylchloride with 2-hydroxybenzyltriphenyl phosphonium bromide in aprotic medium (toluene) in the presence of trimethylamine. In DMF solvent, compounds **7a,b** underwent Mannich reaction with formaline and 5-((piperazin-1-yl)methyl)quinolin-8-ol resulting in 5-((4-((2-(4-substitutedphenyl)benzofuran-3-yl)methyl)piperazin-1-yl)methyl)quinolin-8-ol derivatives (**8a,b**). The structure of the compounds was established on the bases of IR, ^1^H NMR, ^13^C NMR and mass spectral, and elemental analysis data. IR spectra of **7a,b** revealed two characteristic bands at 3130–2956 cm^-1^ due aromatic CH stretching and 885–695 cm^–1^ due to aromatic CH out of plane bending. Conversion of compounds **7a,b** into compounds **8a,b** results in appearance of new characteristic IR bands appeared at 3364–3328 cm^-1^ due to stretching band of the phenolic OH group of the 8-hydroxyquinoline moiety. 1H NMR spectra of compounds **7a,b-8a,b** showed the appearance of 2-phenylbenzofuran signals at δ 6.66–8.68 ppm. Moreover, compounds **7a and 8a** showed characteristic ethyl group triplet-quartet signal pattern at δ 1.25–1.33 ppm and at δ 2.42–2.74 ppm. Compounds **7a and 8b** showed characteristic methoxy group signal at δ 3.82–3.90 ppm and compounds **7f and 8f** revealed characteristic methyl group signal at δ 2.32–2.44 ppm. Compounds **8a-8g** were characterized by the appearance of two signals of the piperazine ring at δ 2.19–2.7 ppm and at δ 2.56–3.05 ppm and appearance of two N-methylene protons signals at δ 3.39–4.04 ppm. 13C NMR spectra of compounds **7a-7g** revealed characteristic signals at δ 99.07–101.48 ppm due to benzofuran-C3 which disappeared in compounds **8a-8g**.

### Thioflavin T (ThT) assay to study Aβ aggregation

**Fig 4:**
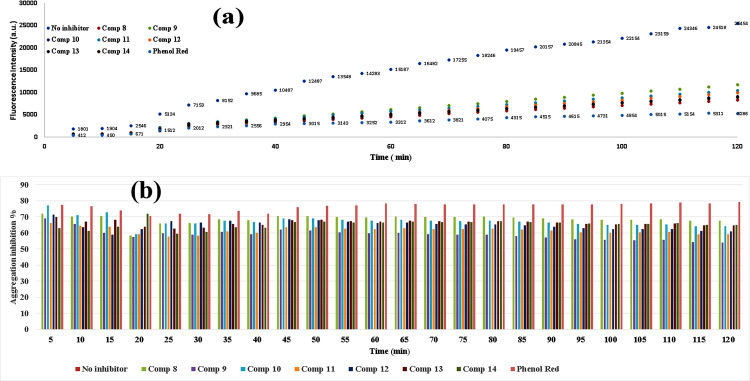
Fluorescence spectrum of bound ThT at the end of the aggregation process with 1 μM Aβ1–42: The interference of compounds 8a-8g with the growth of Aβ1–42 aggregations measured by ThT assay. (a) Fluorescence intensity against time (b) Aggregation inhibition percent versus time.

One of the most widely used benzothiazole derivatives, thioflavin T (ThT), is used as a dye for the *in vitro* and *in vivo* detection and quantification of amyloid beta plaques in the brains of AD patients. To do this, fluorescent spectroscopy is used to assess the fluorescence intensity of bound ThT to amyloid fibrils. This colorful molecule is made up of a benzylamine ring and a benzothiazole ring connected by a single carbon–carbon bond. Two rings can smoothly spin across the single carbon-carbon link and quench the fluorescence intensity when this molecule is activated by a photon in a solution. However, when ThT molecules connect noncovalently with the beta sheets of amyloid fibrils, the two rings become stationary on amyloid aggregations and increase their fluorescence intensity [32]. At doses of 1 μM with thioflavin T (20 μM), the synthetic compounds **8a-8g** were tested for their *in vitro* inhibitory action on Aβ–amyloid aggregation. In Fig 4, the fluorescence spectrum is shown. Thioflavin T assay results indicate that compounds **8a-8g** can inhibit the growth and development of fibrils by reducing the fluorescence of ThT molecules at a wavelength of around 485 nm (Fig 4A). Curcumin and its analogs had the same effects [37]. According to Eq 1, the proportion of Aβ-amyloid peptide aggregation inhibition was estimated (Table 2 and Fig 4B and S4 File). The studied compounds demonstrated encouraging levels of Aβ-peptides aggregation inhibition, ranging from 54.12% to 67.52%. The tested compounds explored comparable inhibition percent values with highest values assigned for compound **8a** and lowest value assigned to compound **8g**.


%inhibition=[1– (TestedRFU–Blank)(maxcontrolRFU–Blank)] ×100 Eq 1

Where Tested RFU is the relative fluorescence units of samples that contain reaction mixtures and tested compounds; Blank RFU is the relative fluorescence units of a solvent sample (which is devoid of both tested compounds and reaction mixtures); and maximum control RFU is relative the fluorescence unit of samples that contain only reaction mixtures (and not test compounds).

### Determination of ligand/iron(III) stoichoimetries using UV/Vis spectrophotometry

**Fig 5:**
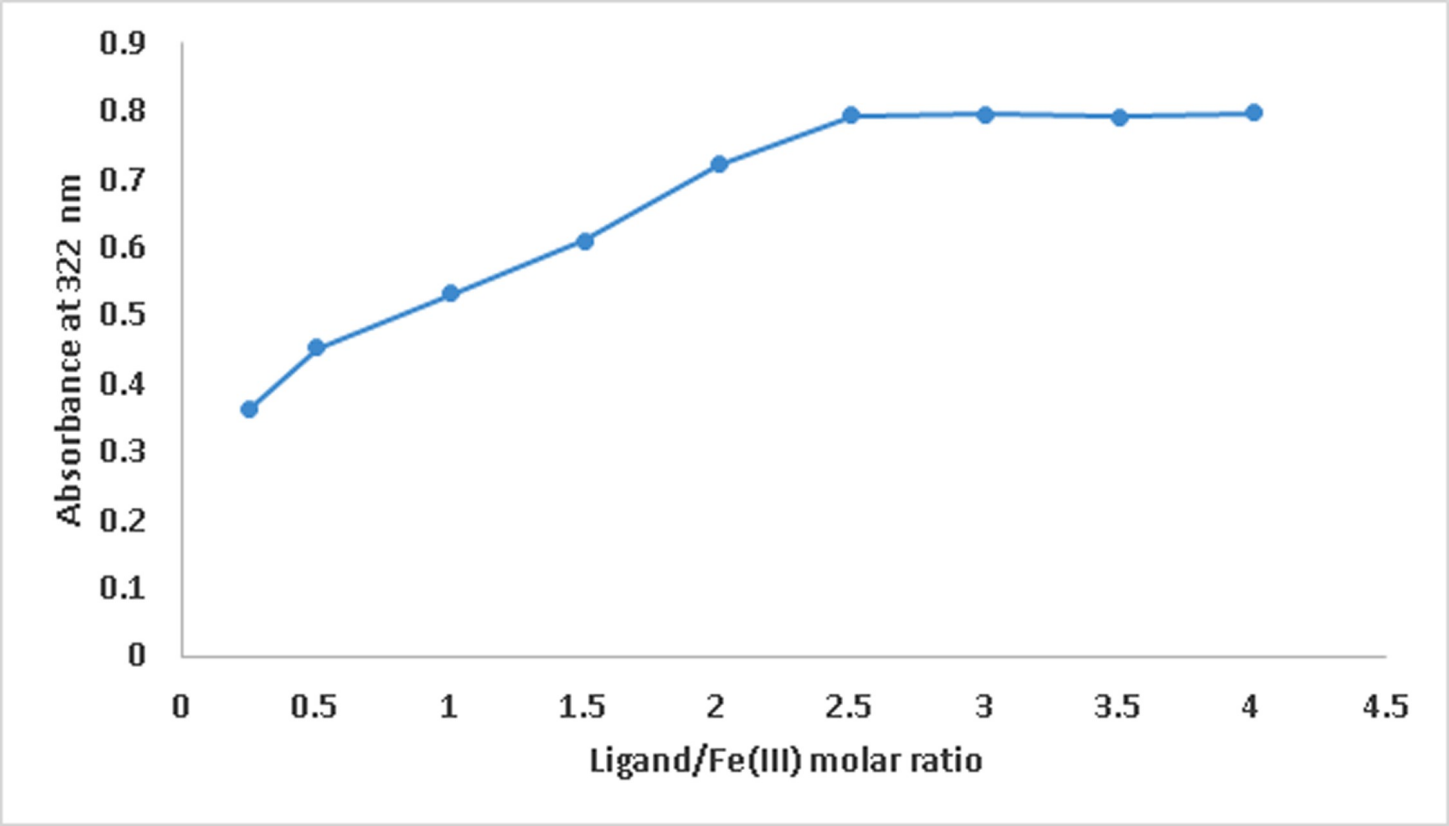
Plot of absorbance at 322 nm versus ligand/iron(III) molar ratios.

The wavelength scan of compound **8e** showed a primary absorption at 307 nm. The band at 307 nm provided a bathochromic shift to longer wavelength 322 nm upon additions of Fe(III) ions to constant concentration of the ligand solution. The absorbance at 322 nm is shown against the ligand/Fe(III) molar ratio in Fig 5. The greatest intensity of the band at 322 nm, attributed to the Fe(III) complex following the addition of 3 mol equivalents of Ligand to the Fe(III) solution, suggests that the iron complex generated has a stoichiometry of 1:3 for Fe(III) and Ligand.

### ADMET prediction

Molecule characteristics such as hydrophobicity, electronic distribution, hydrogen bonding, molecule weight, pharmacophore entity, bioavailability, reactivity, toxicity, and metabolic stability influence the similarity of a medicine. One of the methods most frequently used to determine a compound’s solubility and permeability and, thus, to determine whether or not it is a medication candidate is Lipinski’s rule. The Lipinski rule of five indicates that a molecule is more likely to have poor absorption or penetration if it includes more than five H-bond donors, a molecular weight (MWt) more than 500, a log P value above 5, and more than 10 H-bond acceptors. Table 1 illustrates the drug likeness of the compounds 8a–g. Compounds **8a–d, 8f** values lie within the usual range and their bioavailability scores are 0.55 and they do not all deviate from the Lipinski rule while compounds 8e, 8g have one violation of lipinski’s rule because of its molecular weight values higher than 500. The studied compounds **8a–e** are extensively absorbed via the GI after oral administration, are able to cross the blood–brain barrier (BBB), and can be eliminated by P-glycoprotein (P-gp). Compounds **8g**, on the other hand, are significantly absorbed through GI but are unable to cross the blood–brain barrier. This might be explained by the fact that compound 8g has a greater molar refractivity (MR) value (MR: 169.41) than the other compounds put to the test. Compounds 8a–g have no Brenk or PAINS descriptor alarms, which is another encouraging sign that they could be therapeutic candidates. In medicinal chemistry, the Brenk and pan assay interference compounds (PAINS) structural warnings have been utilized to anticipate the presence of unstable, reactive, and hazardous fragments.

**Table 1:** Physicochemical and pharmacokinetics of the compounds 8a-g using SwissADME server.

Comp.	MW (g/mol)	HBA	HBD	TPSA (Å^2^)	Consensus Log Po/w *	MR	GI Absorption	BBB permeant	P-gp substrate	Lipinski rule	Bioavailability Score	PAINS (alert)	Brenk (alert)
8a	477.60	5	1	52.74	5.06	153.74	High	Yes	Yes	Yes	0.55	0	0
8b	479.57	6	1	61.97	4.44	150.46	High	Yes	Yes	Yes	0.55	0	0
8c	467.53	6	1	52.74	4.75	143.93	High	Yes	Yes	Yes	0.55	0	0
8d	483.99	5	1	52.74	4.99	148.98	High	Yes	Yes	Yes	0.55	0	0
8e	528.44	5	1	52.74	4.94	151.67	High	Yes	Yes	Yes*	0.55	0	0
8f	463.57	5	1	52.74	4.77	148.94	High	Yes	Yes	Yes	0.55	0	0
8g	525.64	5	1	52.74	5.70	169.41	High	No	Yes	Yes*	0.55	0	0

### Molecular docking studies

**Fig 6:**
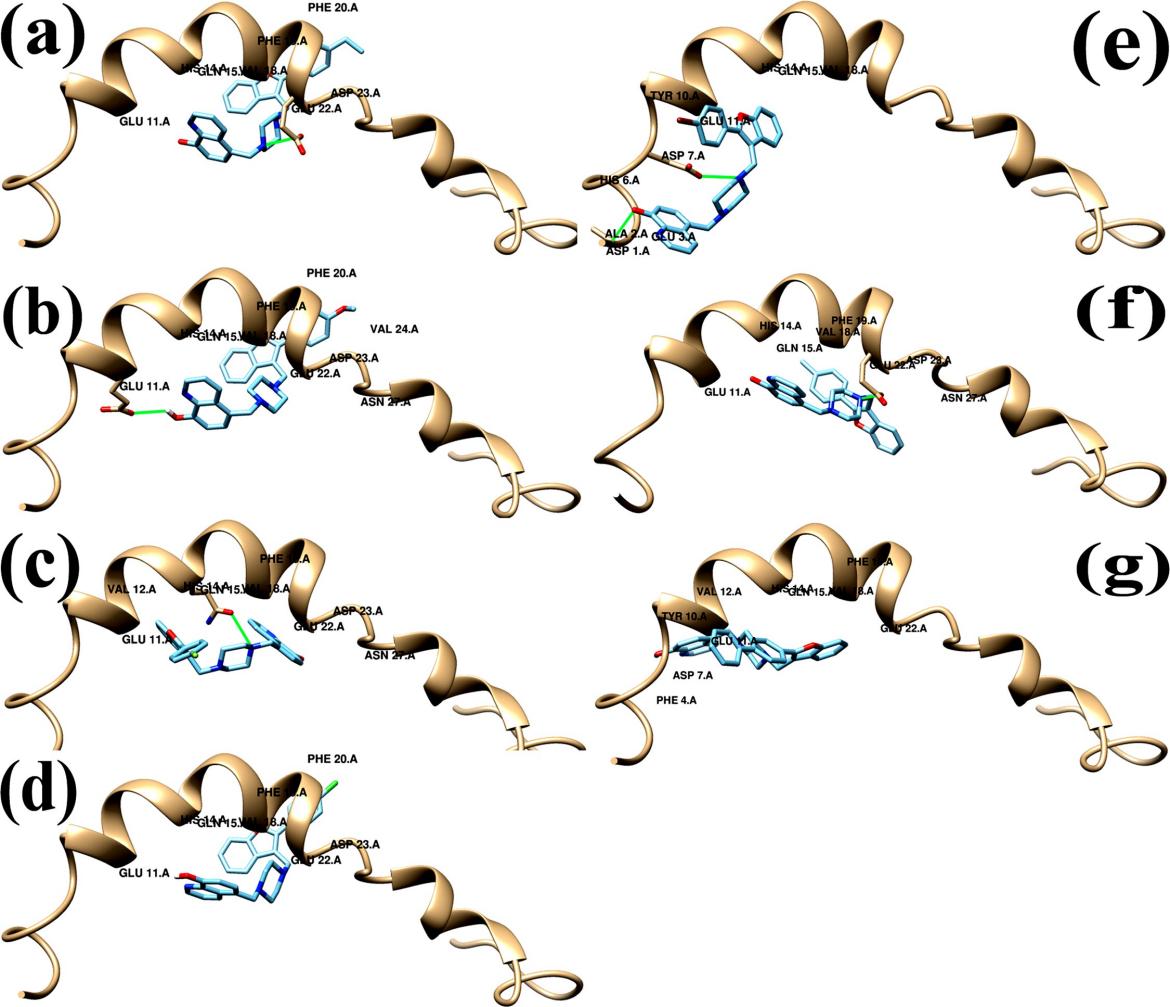
The 3D docked representation of non-covalent interactions between (a) compound 8a, (b) compound 8b, (c) compound 8c, (d) compound 8d, (e) compound 8e, (f) compound 8f, (g) compound 8g and surrounding amino acid residues of Aβ monomer.

**Fig 7:**
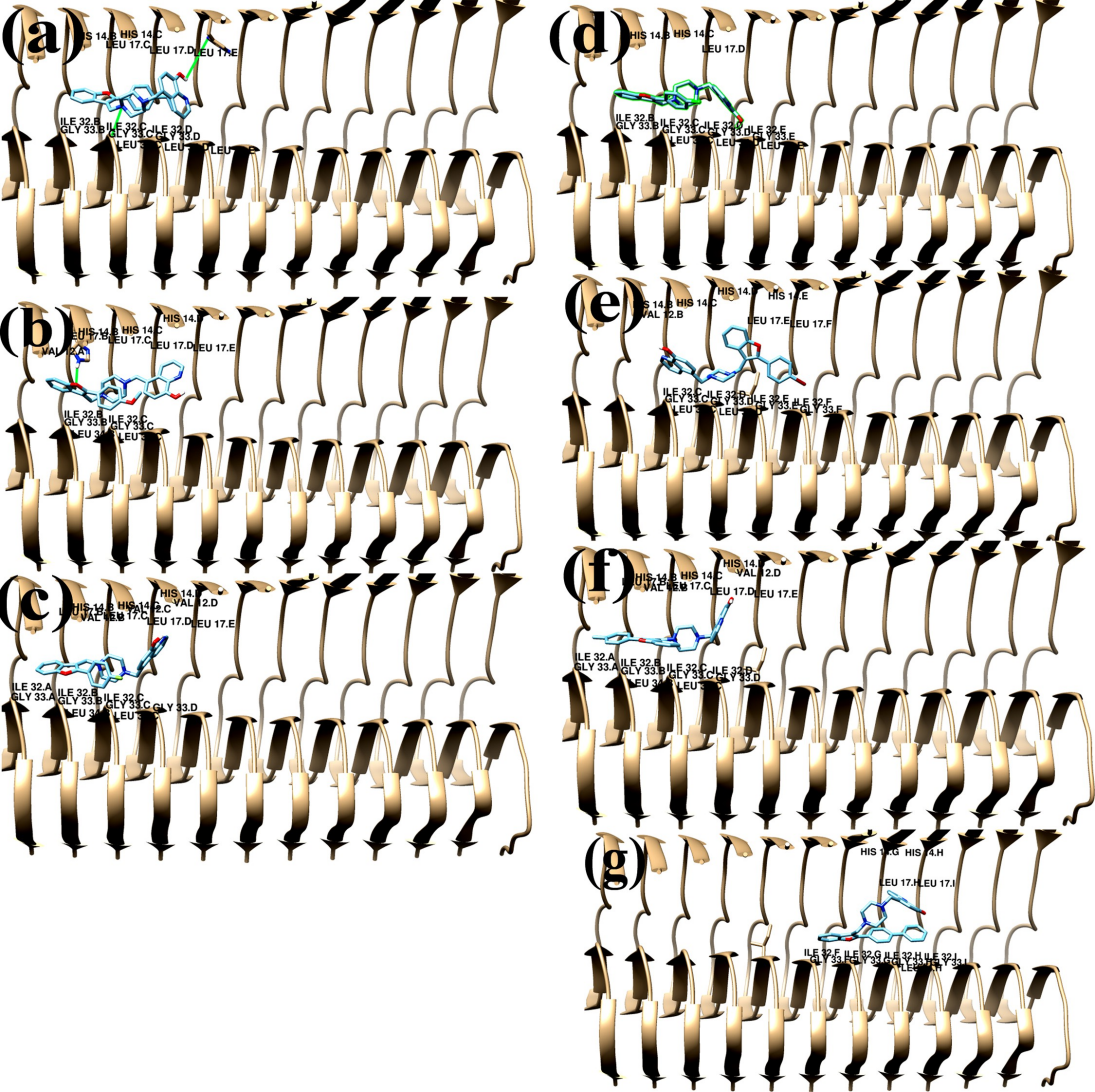
The 3D docked representation of non-covalent interactions between (a) compound 8a, (b) compound 8b, (c) compound 8c, (d) compound 8d, € compound 8e, (f) compound 8f, (g) compound 8g and surrounding amino acid residues of Aβ fibril.

One of the most used computational techniques, molecular docking can illustrate crucial biochemical processes by forecasting the binding locations of ligands to the target [38]. We therefore performed molecular docking studies using AutoDock 4.2 software [36] to determine the molecular interactions and binding affinity between the compounds **8a-8g** and Aβ1–42 monomer (Aβm) and Aβ1–42 fibril (Aβf). The protein database PDB was initially used to obtain the crystal structures of the human Aβ amyloid monomer (Aβ m) [34] and Aβ amyloid fibril (Aβ f) [35]. The synthesized compounds **8a-8g** were drawn, optimized, and transferred to pdbqt format before being docked to the structures of the Aβm and Aβf. The docking study’s findings matched the experimental Aβ1-42-aggregation inhibition percentage. The docking data produced the preliminary estimation of the free binding energies revealing the effectiveness of the ligand–receptor complexed system (Table 2), binding sites, and the residues involved in interactions with the binders **8a-8g** (Table 3 and Figs 6 and 7). The binding free energy score for fibril protein was lower than that for the amyloid monomer, suggesting that these substances can bind to fibrils through positive interaction.

**Table 2:** Experimental Aβ42-aggregation inhibition % and docking results of compounds 8a-8g to Aβ amyloid monomer and Aβ amyloid fibril using AutoDock 4.2 software.

No.	Binding Energy Kcalmol^-1^ Aβm (Aβf)	Inhibition constant (Ki nmol) Aβm (Aβf)	Aβ42-aggregation inhibition %
**8a**	-8.79 (-9.85)	358.06 (327.42)	67.52
**8b**	-7.78 (-8.46)	364.7 (628.88)	54.12
**8c**	-8.57 (-8.92)	526.07 (290.92)	64.10
**8d**	-8.35 (-8.85)	139.93 (323.52)	59.25
**8e**	-9.73 (-8.98)	73.29 (259.95)	61.00
**8f**	-8.56 (-8.84)	530.08 (331.25)	64.64
**8g**	-8.92 (-9.98)	53.58 (48.02)	65.04

For the complexes of compounds 8a-Aβm, 8b-Aβm, 8c-Aβm, 8d-Aβm, 8e-Aβm; 8f-Aβm and 8g-Aβm docking has calculated the binding energy to be -8.79, -7.78, -8.57, -8.35, -9.73, -8.56 and -8.92 kcal/mol, respectively. When the ligands are coupled to Aβm via non-covalent interactions to form stable complexes, the docking analysis predicted the energetically advantageous poses of the ligands in the binding site, the central hydrophobic area (Lys16 to Ala21), of the Aβm (Table 3). In these bound complexes, the development of hydrogen bonds and hydrophobic interactions were significant factors (Fig 6). The acquired docked poses of the ligands in the binding cavity revealed the hydrogen-bonding residues to the ligands, the core hydrophobic area amino acid residues 14–23 of the Aβm that is essential for monomer-monomer interactions, and the aggregation of the Aβ peptide into the Aβ fibril [28, 39]. A critical nucleation location for amino acids, region Aβ16–20, interacts with homologous regions 17–21 or 18–22 to form an antiparallel β–sheet structure [40]. Aβ fibril production has reportedly been inhibited by ligands that bind to the Aβ-16–20 region of the amyloid monomer [41]. The mechanism of Aβ1–42 aggregation and fibrillation depends heavily on ligands that target residues close to the hydrophobic cluster (17–21) [41]. Tao et al. (2011) [41] showed that the favonoids inhibited Aβ1–42 aggregation by specifically targeting the Lys residues because Lys16 close to the hydrophobic cluster (17–21) was essential for Aβ aggregation and its toxicity. Thus, the usefulness of the positively interacting compounds **8a-8g** in capping the monomer’s nucleation site has been demonstrated. 

**Table 3:** Interacting amino acid residues of amyloid monomer with compounds 8a-8g.

No	Binding residues	Hydrogen bonds (distance, Å)
**8a**	Glu11, His14, Gln15,Val18, Phe19,Phe20, Glu22, Asp23	Glu22 (3.211 Å)
**8b**	Glu11, His14, Gln15,Val18, Phe19,Phe20, Glu22, Asp23, Val24, Asn27	Glu11 (2.734 Å)
**8c**	Glu11, Val12, His14, Gln15,Val18, Phe19, Glu22, Asp23, Asn27	Gln15 (3.015 Å)
**8d**	Glu11, His14, Gln15,Val18, Phe19,Phe20, Glu22, Asp23	No Hb
**8e**	Asp1, Ala2, Glu3, His6, Asp7, Tyr10, Glu11, His14, Gln15,Val18, Phe19	Ala2 (1.797 Å), Asp7 (3.263Å)
**8f**	Glu11, His14, Gln15,Val18, Phe19, Phe20, Glu22, Asp23, Asn27	Glu22 (2.786 Å)
**8g**	Phe4, Asp7, Tyr10, Glu11, His14, Gln15,Val18, Phe19, Phe20, Glu22	Glu11 (2.765 Å)

The calculated binding energy (kcal/mol) of Aβf-compounds (**8a-8g)** complexes was 8a-Aβm, 8b-Aβm, 8c-Aβm, 8d-Aβm, 8e-Aβm; 8f-Aβm and 8g-Aβm docking has calculated the binding energy to be -9.85, -8.46, -8.92, -8.85, -8.98, -8.84 and -9.98. The docked complexes exhibited the non-covalent interactions of compounds **8a-8g** with the residues of the central region: His14, Leu17 and C-terminal regions: Ile32, Gly33, Leu34 of chain B, C, D and E of Aβf-compounds(8a,8c,8d); of chain A, B, C, D and E of Aβf-compounds(8b, 8f); of chain B,C,D,E and F of Aβf-compound(8e); of chain F, G,H, and I of Aβf-compound(8g) (Table 4 and Fig 7). Similar to the reported findings [38, 42], the hydrogen bonds and hydrophobic interactions with these important residues disturb specifically the stable fibrillar structure occurring inside the chains. 

**Table 4:** Interacting amino acid residues of amyloid fibril with compounds 8a-8g.

No	Binding residues
**8a**	Chain B: His14, Ile32, Gly33
	Chain C: His14, Leu17, Ile32, Gly33, Leu34
	Chain D: Leu17, Ile32, Gly33, Leu34
	Chain E: Leu17, Leu34
**8b**	Chain A: Val12
	Chain B: His14, Leu17, Ile32, Gly33, Leu34
	Chain C: His14, Leu17, Ile32, Gly33, Leu34
	Chain D: His14, Leu17
	Chain E: Leu17
**8c**	Chain B: His14, Ile32, Gly33
	Chain C: His14, Ile32, Gly33, Leu34
	Chain D: Leu17, Ile32, Gly33, Leu34
	Chain E: Ile32, Gly33, Leu34
**8d**	Chain B: His14, Ile32, Gly33
	Chain C: His14, Ile32, Gly33, Leu34
	Chain D: Leu17, Ile32, Gly33, Leu34
	Chain E: Ile32, Gly33, Leu34
**8e**	Chain B: Val12, His14
	Chain C: His14, Ile32, Gly33, Leu34
	Chain D: His14, Ile32, Gly33, Leu34
	Chain E: His14, Leu17, Ile32, Gly33
	Chain F: Leu17, Ile32, Gly33
**8f**	Chain A: Ile32, Gly33
	Chain B: Val12, His14, Leu17, Ile32, Gly33, Leu34
	Chain C: His14, Leu17, Ile32, Gly33, Leu34
	Chain D: Val12, His14, Leu17, Ile32, Gly33
	Chain E: Leu17
**8g**	Chain F: Ile32, Gly33
	Chain G: His14, Ile32, Gly33
	Chain H: His14, Leu17, Ile32, Gly33, Leu34
	Chain I: Leu17, Ile32, Gly33

## Conclusions

The work included the design and synthesis of new small molecules as hybrids of benzofuran and 8-hydroxyquinoline moieties that are linked together through piperazine spacer. The compounds were proposed as potential multi-target anti-Alzheimer’s agents that combine the anti-fibrillation activity of benzofuran scaffold, the antimuscarinic activity of piperazine ring and the iron-chelation properties of 8-hydroxyquinoline moiety. The synthesized compounds exhibited a significant reduction of about 54.12%-76.59% against the Aβ42 aggregation using ThT assay that also supposed a good correlation between docking study and experimental methods. In conclusion, the favorable characteristics of our newly synthesized compounds make it promising candidates β-amyloid-fibrillation inhibitors for further development and studies.

## Supporting information

S1 File Figs 1–7: 1H NMR spectrum of compounds 7a-7g. (PDF)

S2 File Figs 1–7 1H NMR spectrum of compounds 8a-8g. (PDF)

S3 File Figs 1, 2 1H NMR and 13CNMR of compound 5. (PDF)

S4 File Table 1 Thiophlafin assay results. (XLSX)
