# Supplementary material for: Design and synthesis of multi-functional small-molecule based inhibitors of amyloid-β aggregation: Molecular modeling and in vitro evaluation
Source: PLoS One. 2023 May 25;18(5):e0286195. doi: 10.1371/journal.pone.0286195 (PMC10212088; doi:10.1371/journal.pone.0286195)

PROTON CDCl3 {C:\Bruker\TOPSPIN} abari 11

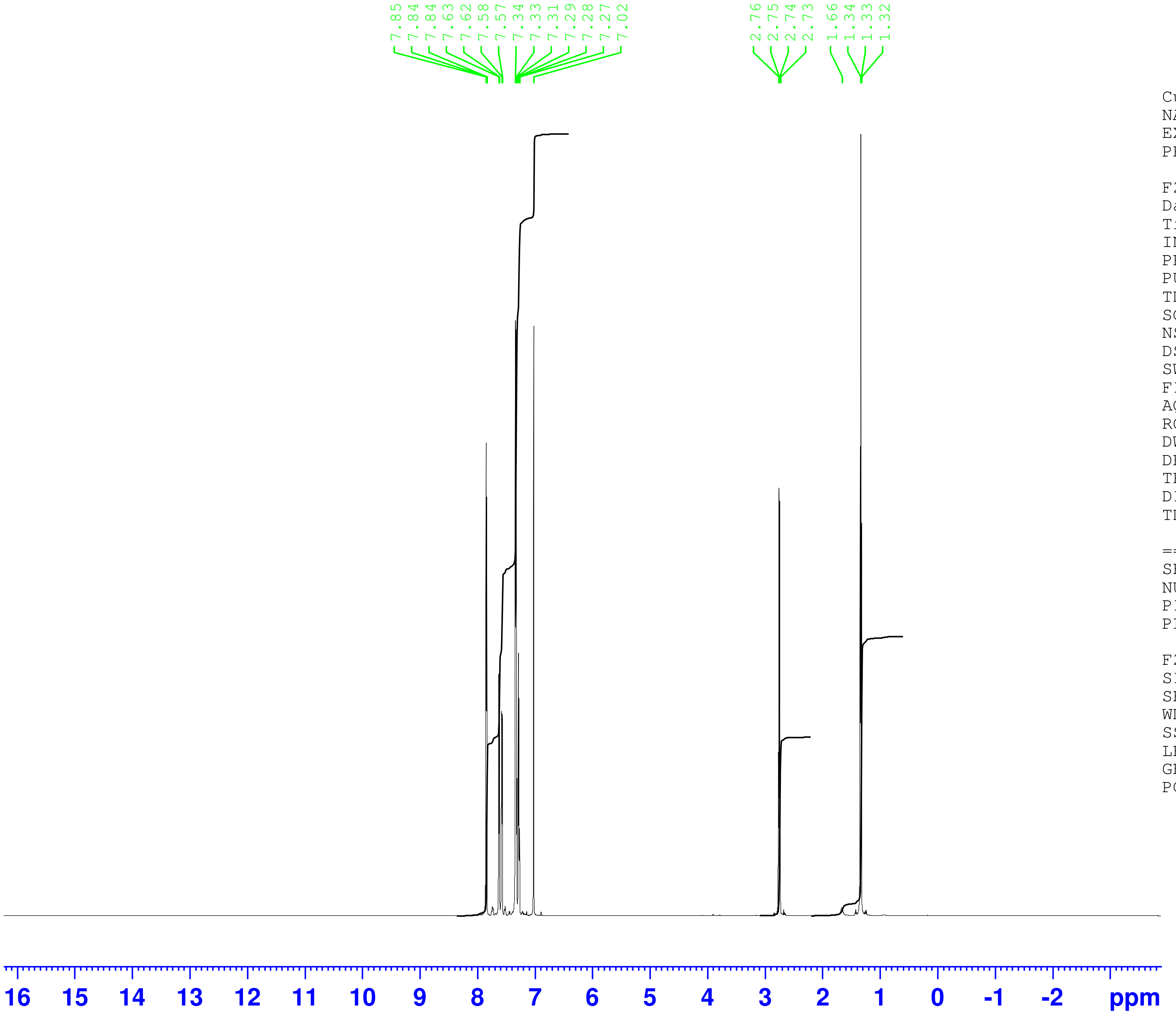

Current Data Parameters
NAME drSalama-F-1a
EXPNO 10
PROCNO 1

F2 - Acquisition Parameters
Date_ 20201124
Time 1.14
INSTRUM spect
PROBHD 5 mm CPTCI 1H-
PULPROG zg30
TD 65536
SOLVENT CDCl3
NS 16
DS 2
SWH 14097.744 Hz
FIDRES 0.215115 Hz
AQ 2.3243434 sec
RG 11.14
DW 35.467 usec
DE 31.86 usec
TE 298.0 K
D1 1.00000000 sec
TD0 1

======== CHANNEL f1 ========
SFO1 700.1743238 MHz
NUC1 1H
P1 8.00 usec
PLW1 9.64999962 W

F2 - Processing parameters
SI 65536
SF 700.1700000 MHz
WDW EM
SSB 0
LB 0.30 Hz
GB 0
PC 1.00

PROTON CDCl3 {C:\Bruker\TOPSPIN} abari 7

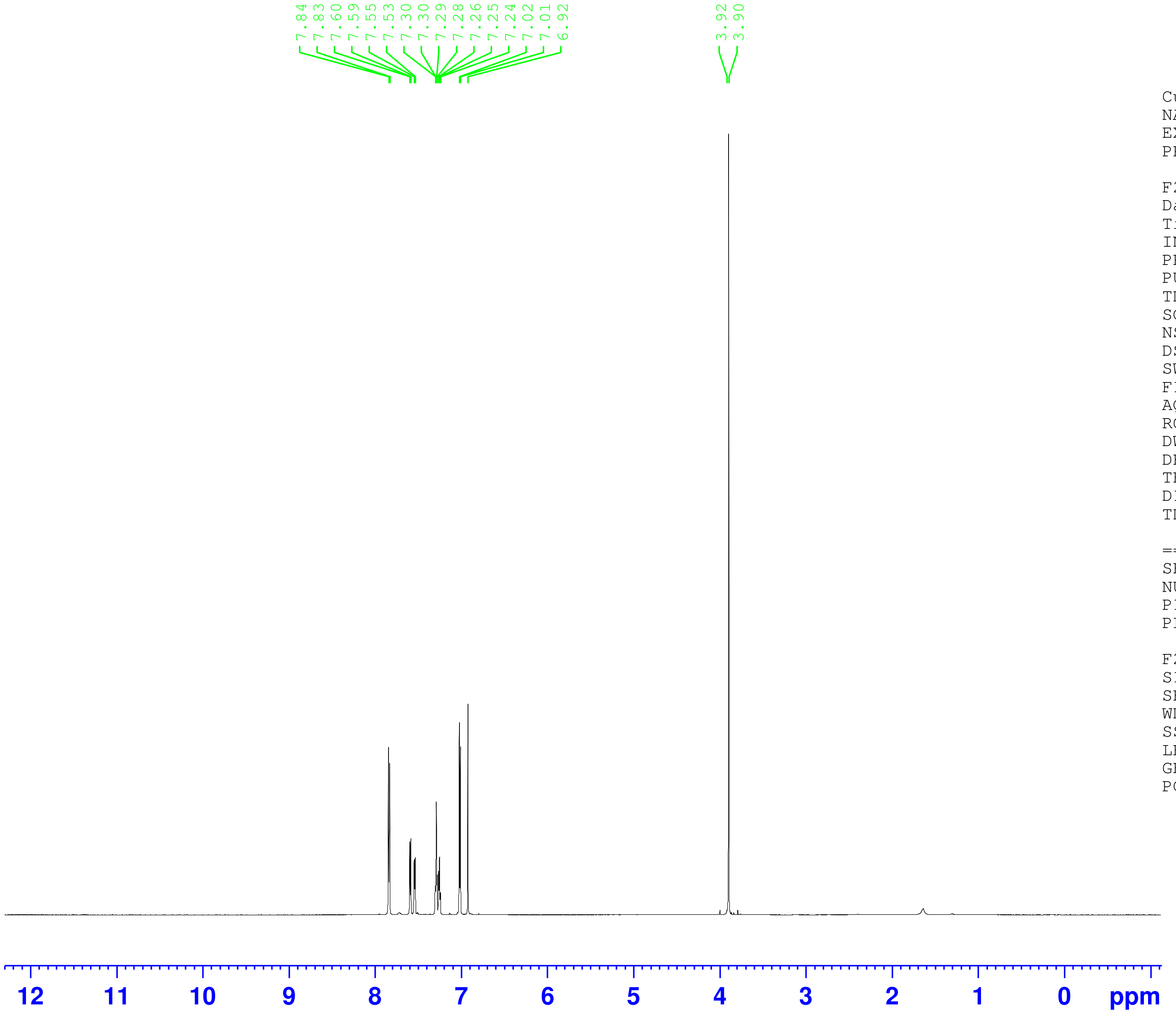

Current Data Parameters
NAME drSalama-F-2a
EXPNO 10
PROCNO 1

F2 - Acquisition Parameters
Date_ 20201123
Time 21.00
INSTRUM spect
PROBHD 5 mm CPTCI 1H-
PULPROG zg30
TD 65536
SOLVENT CDCl3
NS 16
DS 2
SWH 14097.744 Hz
FIDRES 0.215115 Hz
AQ 2.3243434 sec
RG 31.35
DW 35.467 usec
DE 31.86 usec
TE 298.0 K
D1 1.00000000 sec
TD0 1

======== CHANNEL f1 ========
SFO1 700.1743238 MHz
NUC1 1H
P1 8.00 usec
PLW1 9.64999962 W

F2 - Processing parameters
SI 65536
SF 700.1700000 MHz
WDW EM
SSB 0
LB 0.30 Hz
GB 0
PC 1.00

PROTON CDCl3 {C:\Bruker\TOPSPIN} abari 9

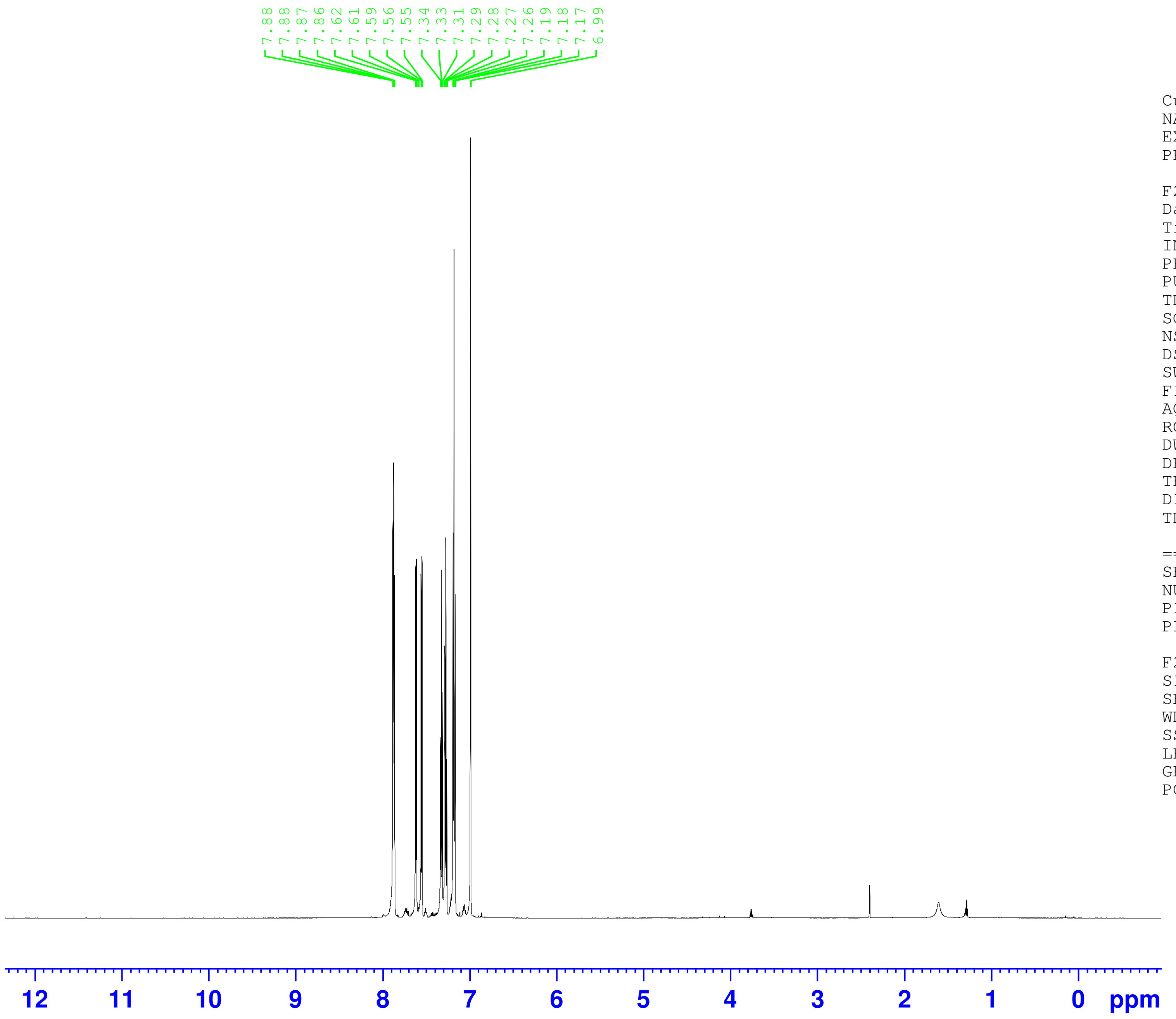

Current Data Parameters
NAME drSalama-F-3b
EXPNO 10
PROCNO 1

F2 - Acquisition Parameters
Date_ 20201123
Time 23.07
INSTRUM spect
PROBHD 5 mm CPTCI 1H-
PULPROG zg30
TD 65536
SOLVENT CDCl3
NS 16
DS 2
SWH 14097.744 Hz
FIDRES 0.215115 Hz
AQ 2.3243434 sec
RG 31.35
DW 35.467 usec
DE 31.86 usec
TE 298.0 K
D1 1.00000000 sec
TD0 1

======== CHANNEL f1 ========
SFO1 700.1743238 MHz
NUC1 1H
P1 8.00 usec
PLW1 9.64999962 W

F2 - Processing parameters
SI 65536
SF 700.1700000 MHz
WDW EM
SSB 0
LB 0.30 Hz
GB 0
PC 1.00

PROTON CDCl3 {C:\Bruker\TOPSPIN} abari 10

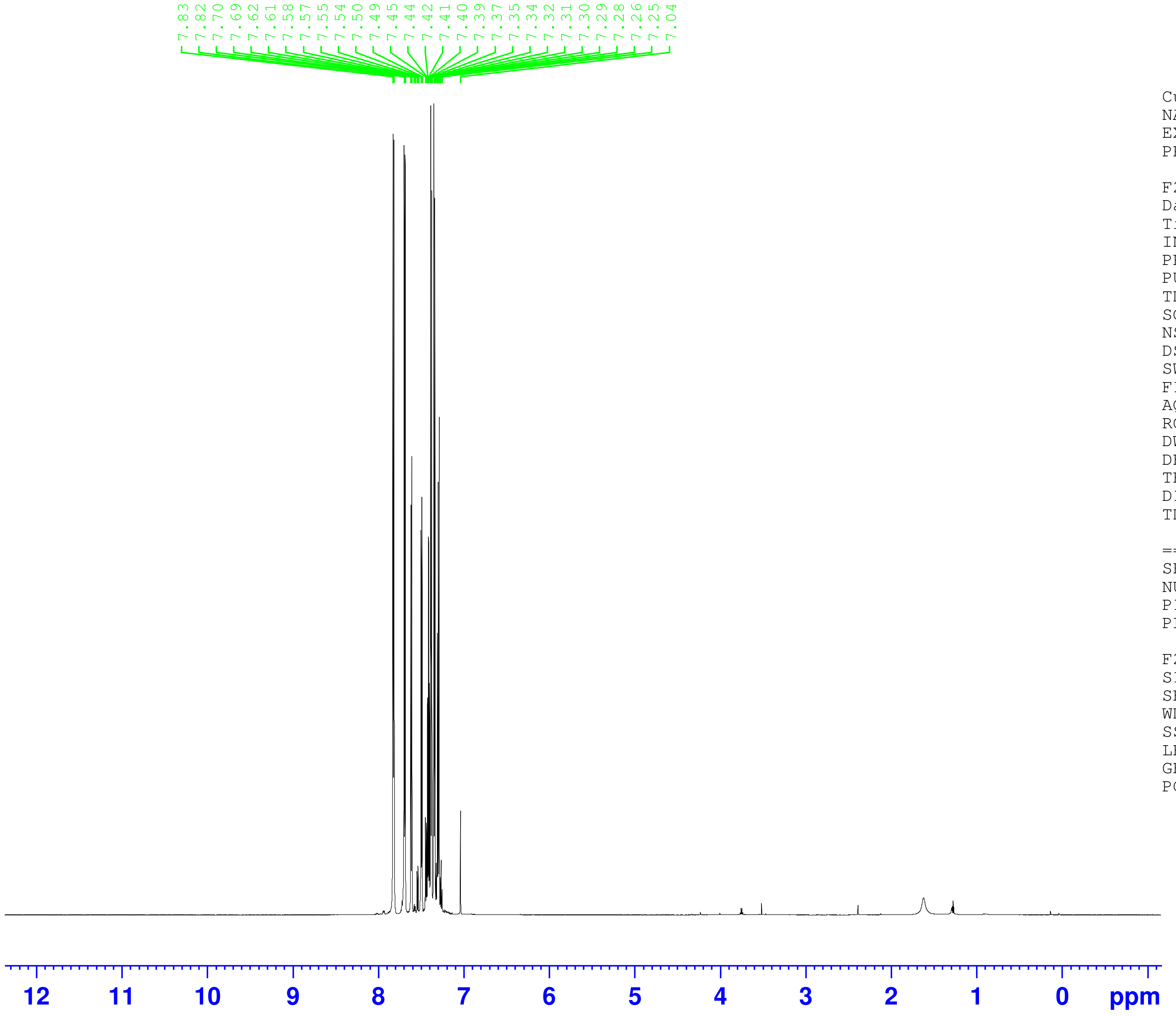

Current Data Parameters
NAME drSalama-F-4b
EXPNO 10
PROCNO 1

F2 - Acquisition Parameters
Date_ 20201124
Time 0.11
INSTRUM spect
PROBHD 5 mm CPTCI 1H-
PULPROG zg30
TD 65536
SOLVENT CDCl3
NS 16
DS 2
SWH 14097.744 Hz
FIDRES 0.215115 Hz
AQ 2.3243434 sec
RG 31.35
DW 35.467 usec
DE 31.86 usec
TE 298.0 K
D1 1.00000000 sec
TD0 1

======== CHANNEL f1 ========
SFO1 700.1743238 MHz
NUC1 1H
P1 8.00 usec
PLW1 9.64999962 W

F2 - Processing parameters
SI 65536
SF 700.1700000 MHz
WDW EM
SSB 0
LB 0.30 Hz
GB 0
PC 1.00

PROTON CDCl3 {C:\Bruker\TOPSPIN} abari 6

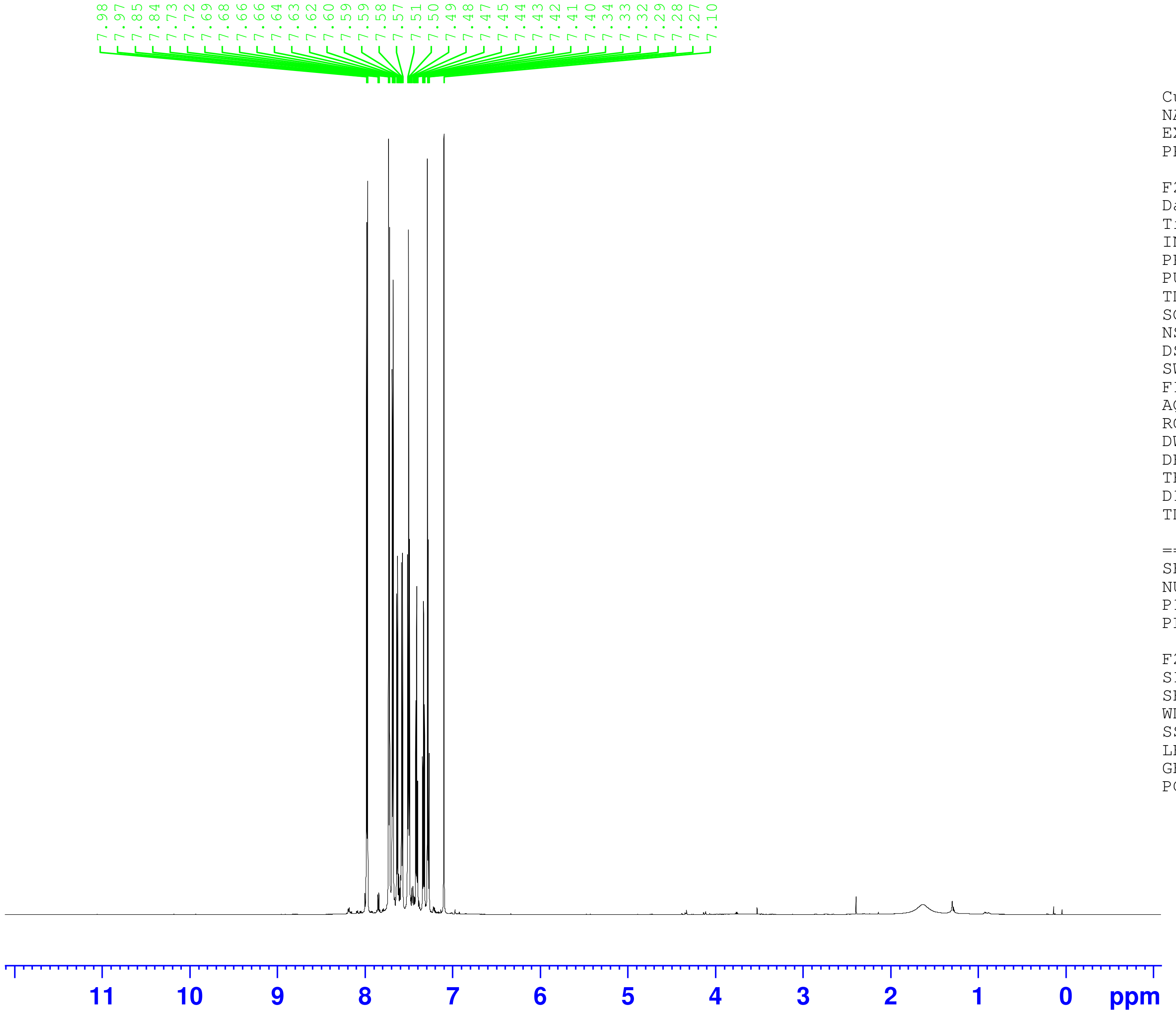

Current Data Parameters
NAME drSalama-F-5
EXPNO 10
PROCNO 1

F2 - Acquisition Parameters
Date_ 20201123
Time 19.56
INSTRUM spect
PROBHD 5 mm CPTCI 1H-
PULPROG zg30
TD 65536
SOLVENT CDCl3
NS 16
DS 2
SWH 14097.744 Hz
FIDRES 0.215115 Hz
AQ 2.3243434 sec
RG 31.35
DW 35.467 usec
DE 31.86 usec
TE 298.0 K
D1 1.00000000 sec
TD0 1

======== CHANNEL f1 ========
SFO1 700.1743238 MHz
NUC1 1H
P1 8.00 usec
PLW1 9.64999962 W

F2 - Processing parameters
SI 65536
SF 700.1700000 MHz
WDW EM
SSB 0
LB 0.30 Hz
GB 0
PC 1.00

PROTON CDCl3 {C:\Bruker\TOPSPIN} abari 8

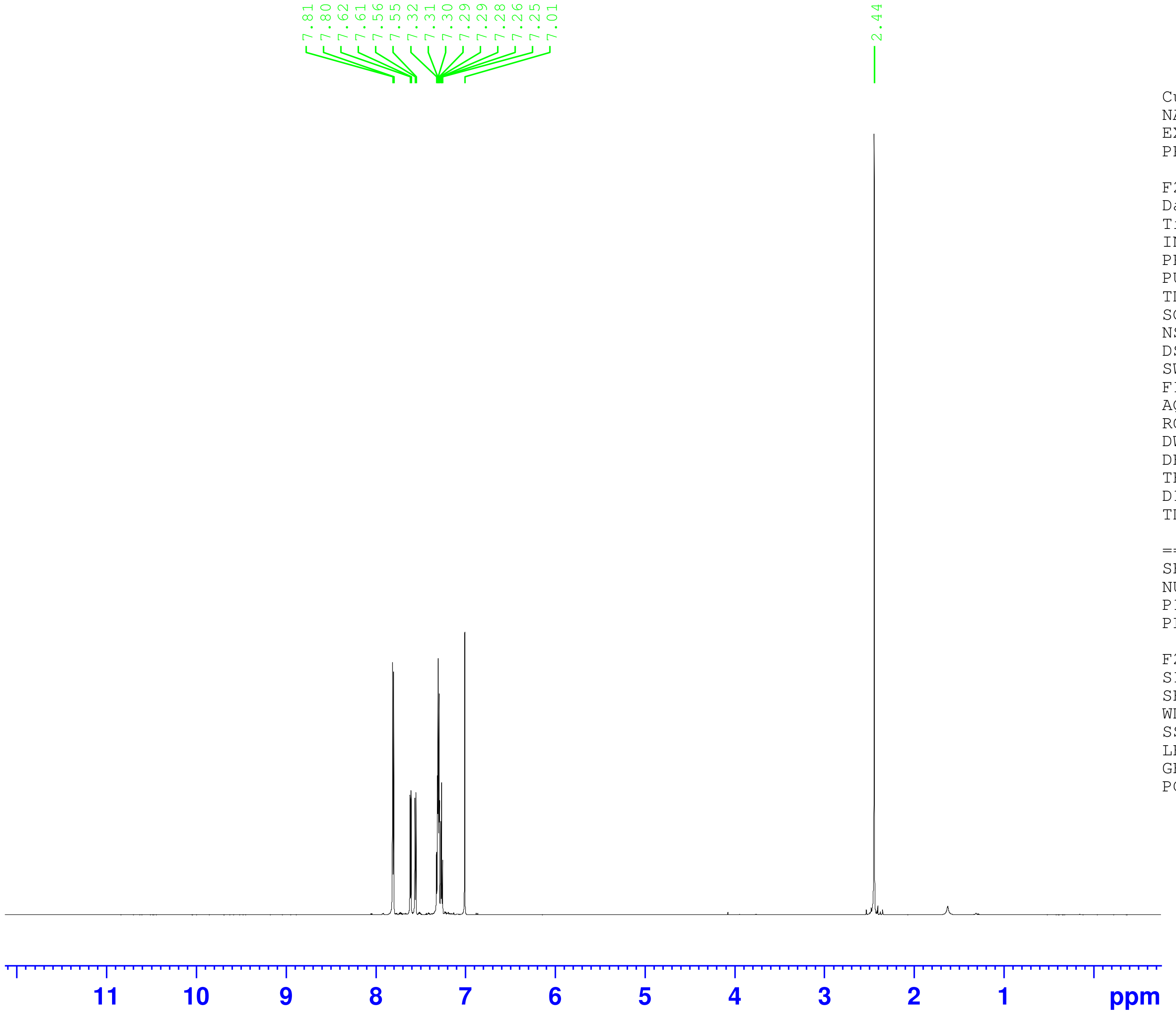

Current Data Parameters

NAME	drSalama-F-6a
EXPNO	10
PROCNO	1

F2 - Acquisition Parameters

Date_	20201123
Time	22.03
INSTRUM	spect
PROBHD	5 mm CPTCI 1H-
PULPROG	zg30
TD	65536
SOLVENT	CDCl3
NS	16
DS	2
SWH	14097.744 Hz
FIDRES	0.215115 Hz
AQ	2.3243434 sec
RG	31.35
DW	35.467 usec
DE	31.86 usec
TE	298.0 K
D1	1.00000000 sec
TD0	1

======== CHANNEL f1 ========

SFO1	700.1743238 MHz
NUC1	1H
P1	8.00 usec
PLW1	9.64999962 W

F2 - Processing parameters

SI	65536
SF	700.1700000 MHz
WDW	EM
SSB	0
LB	0.30 Hz
GB	0
PC	1.00

drSalama-Z-61 10 1 "E:\Work\kayyli_chair\npst_2019_alzheimer\experiment\Analysis_data\salama (2)\salama"

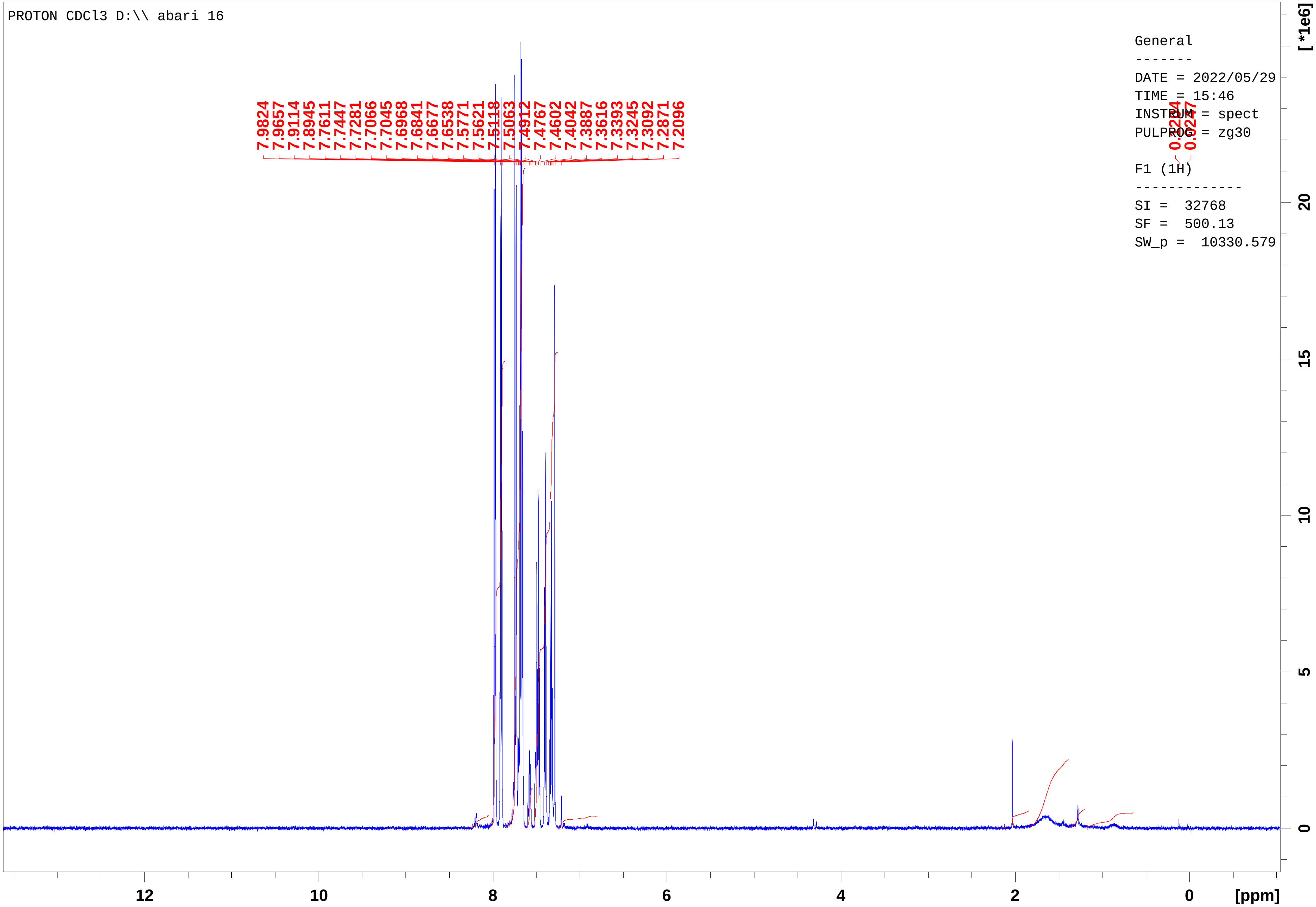

Supplement: S1 File — (PDF) [file pone.0286195.s001.pdf]
