# Supplementary figures and images for: Design and synthesis of multi-functional small-molecule based inhibitors of amyloid-β aggregation: Molecular modeling and in vitro evaluation
Source: PLoS One. 2023 May 25;18(5):e0286195. doi: 10.1371/journal.pone.0286195 (PMC10212088; doi:10.1371/journal.pone.0286195)

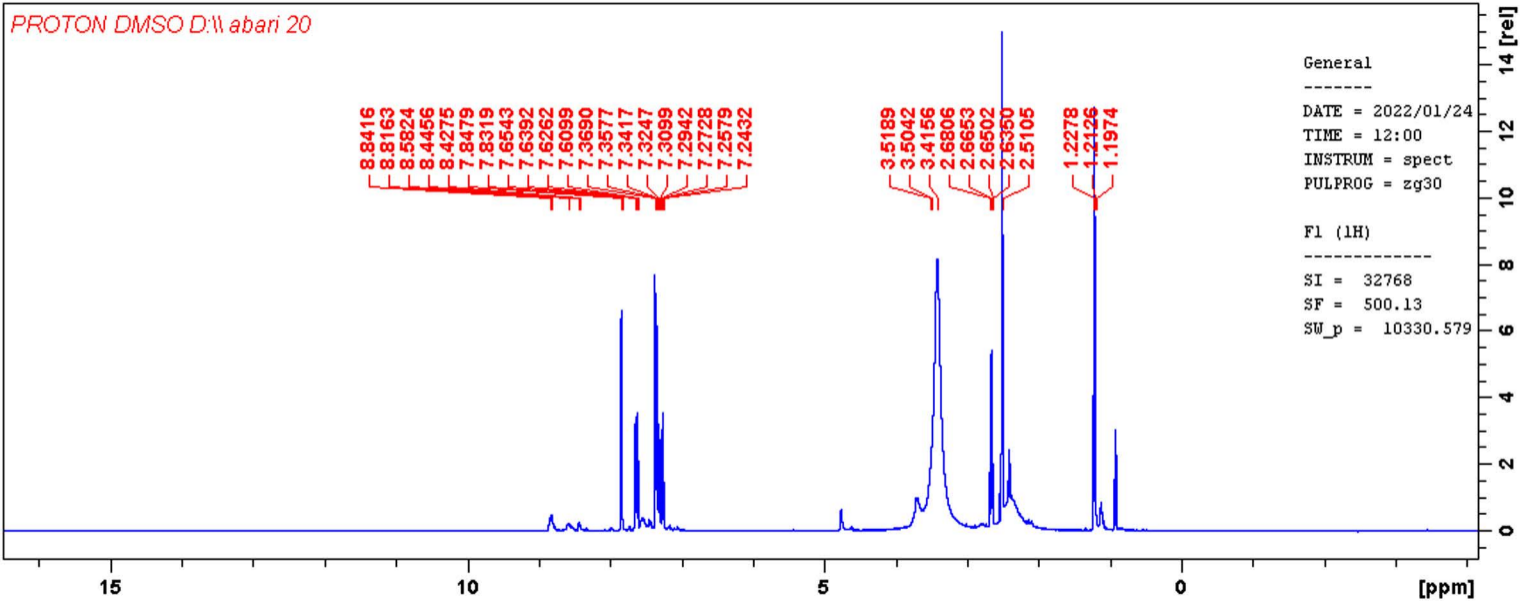

PROTON DMSO D:\ abari 47

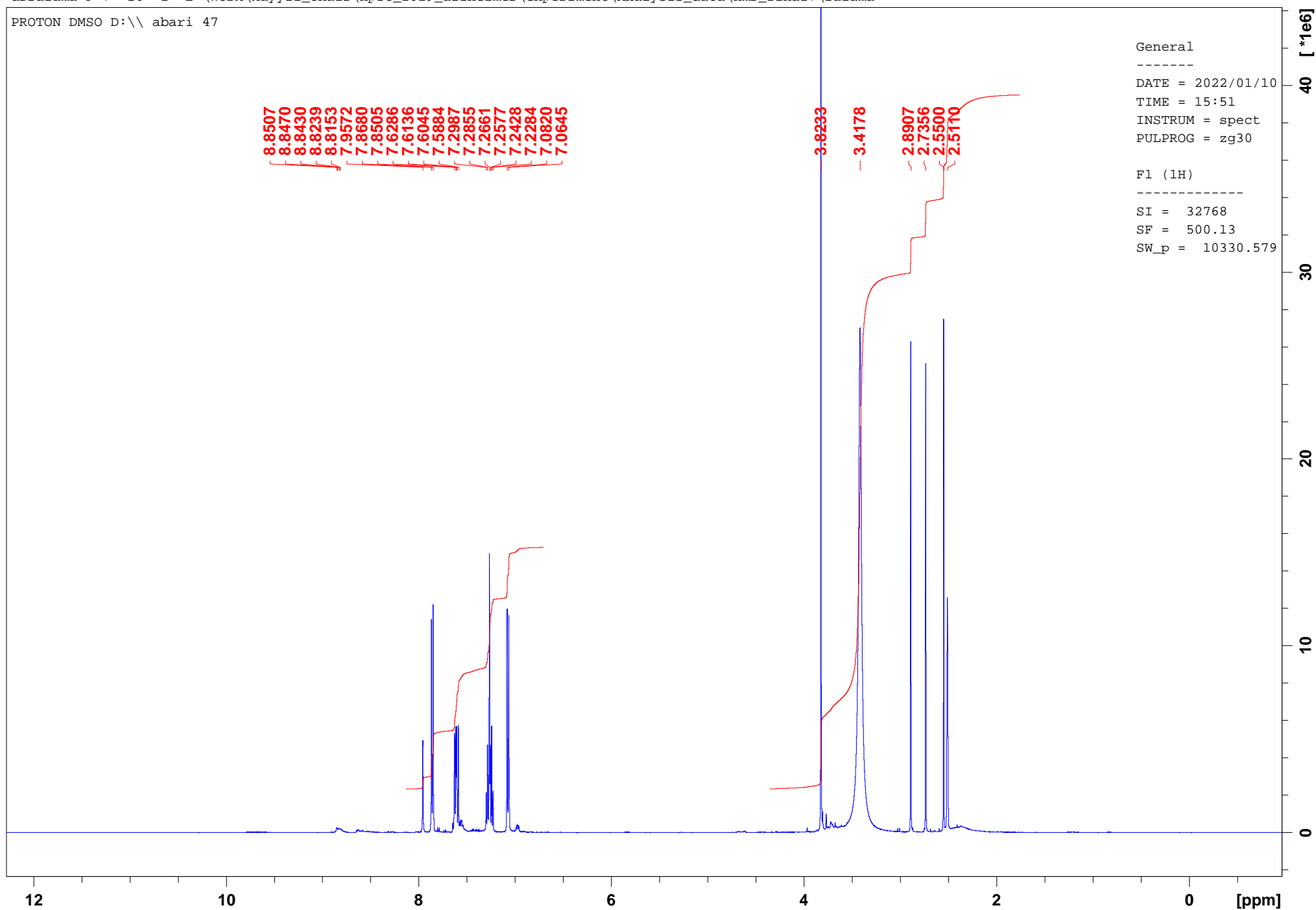

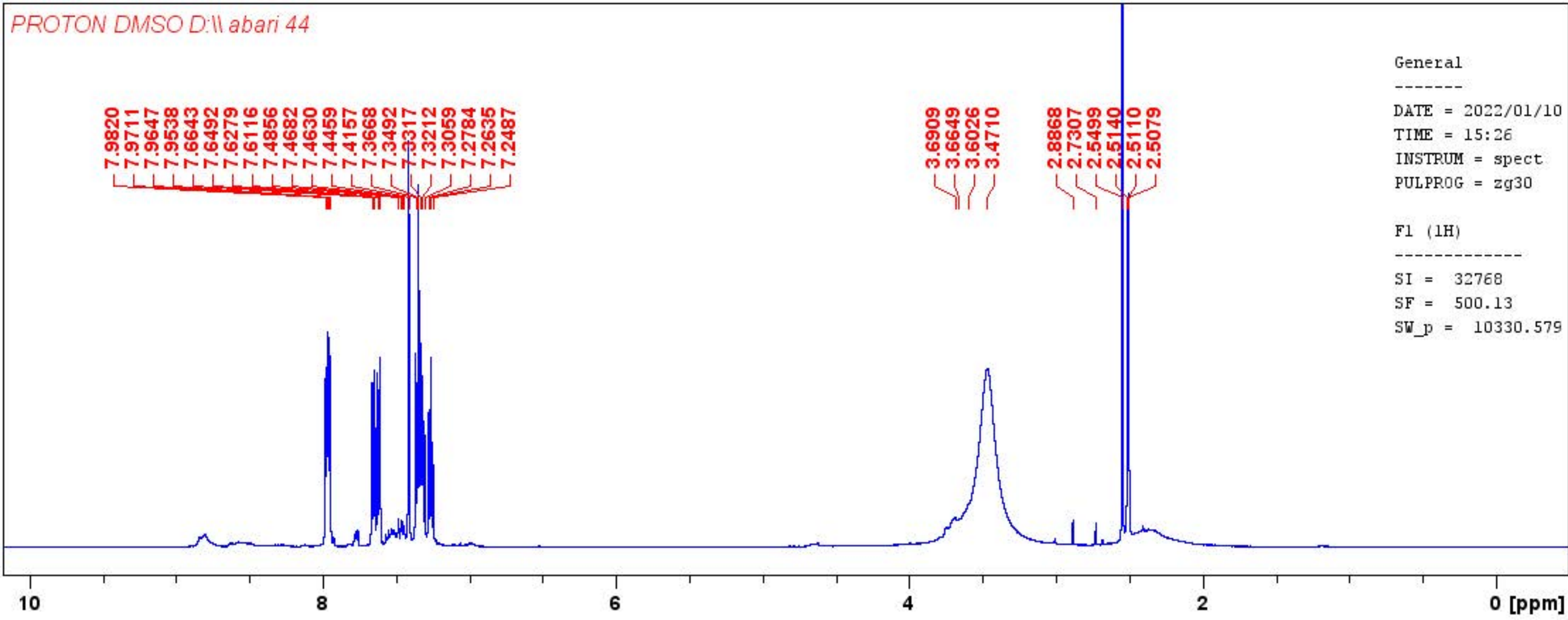

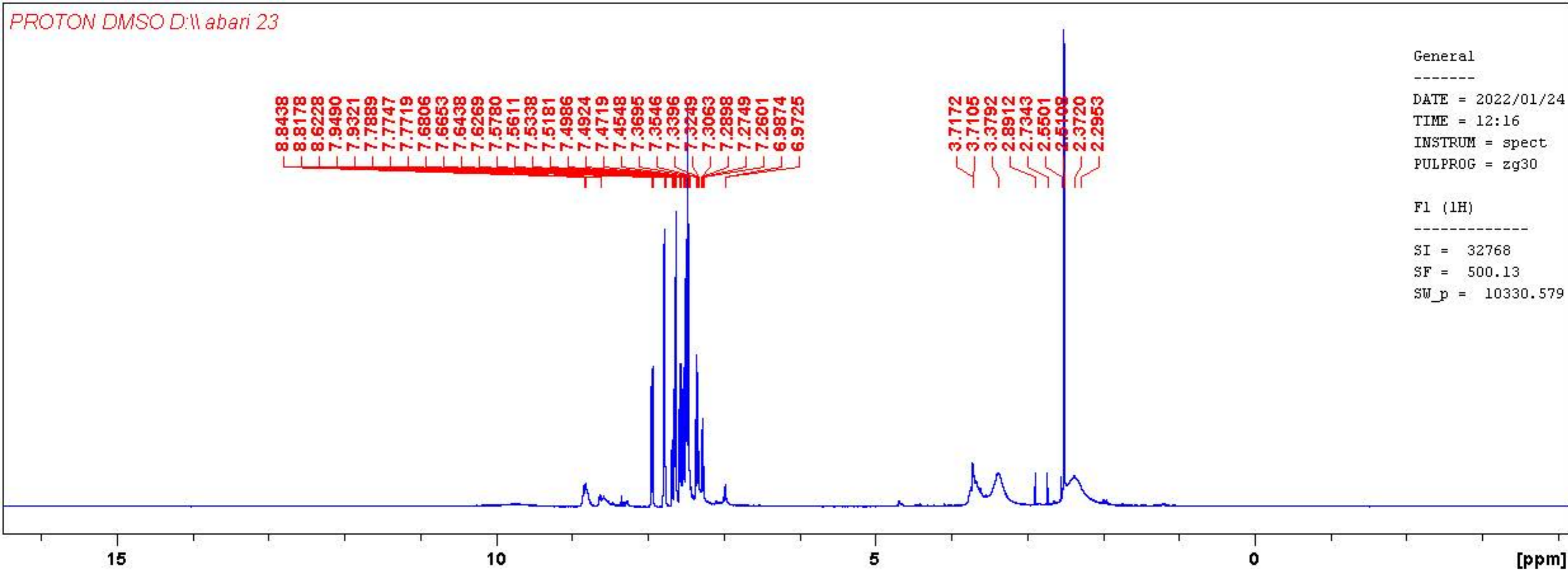

PROTON DMSO D:\ abari 47

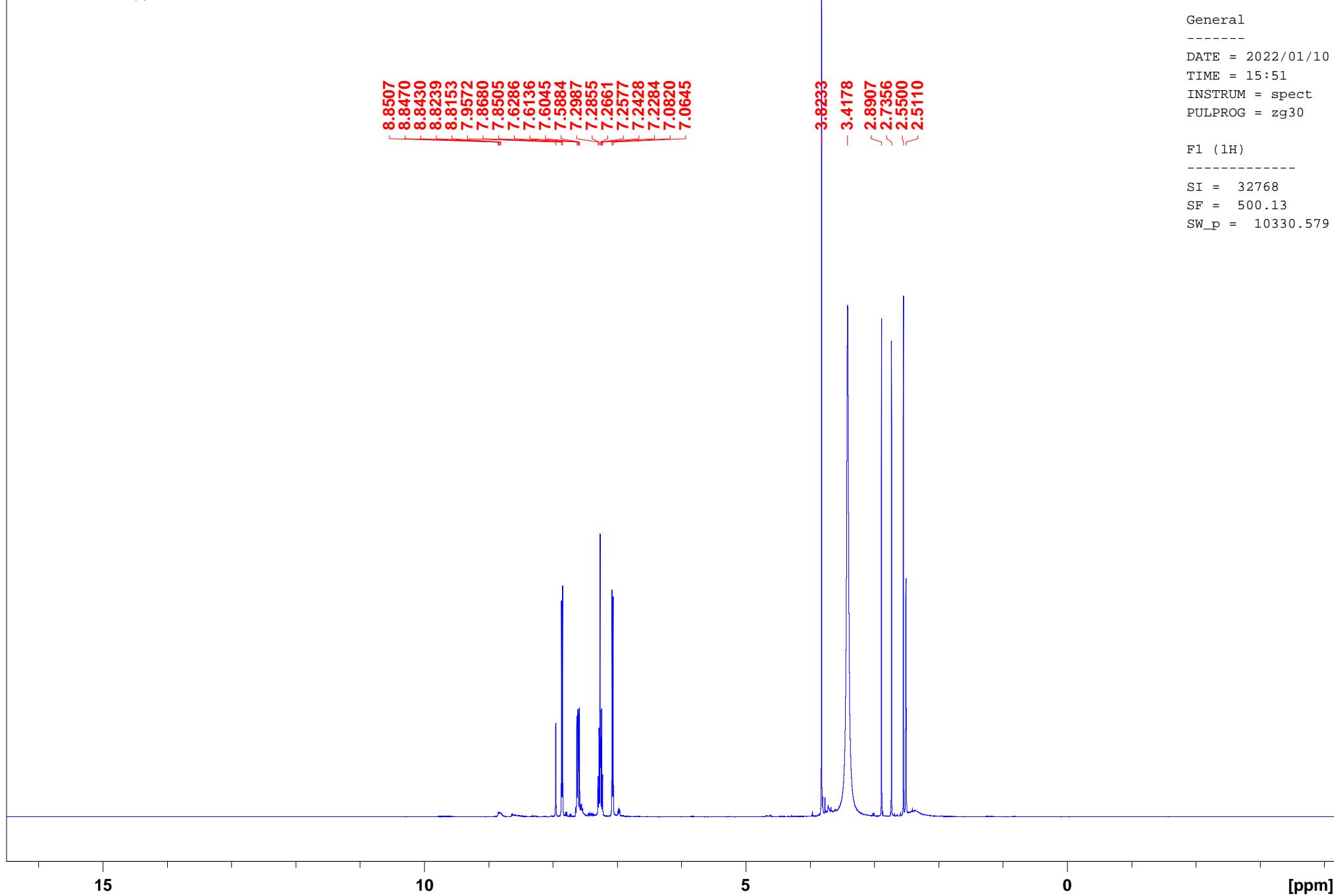

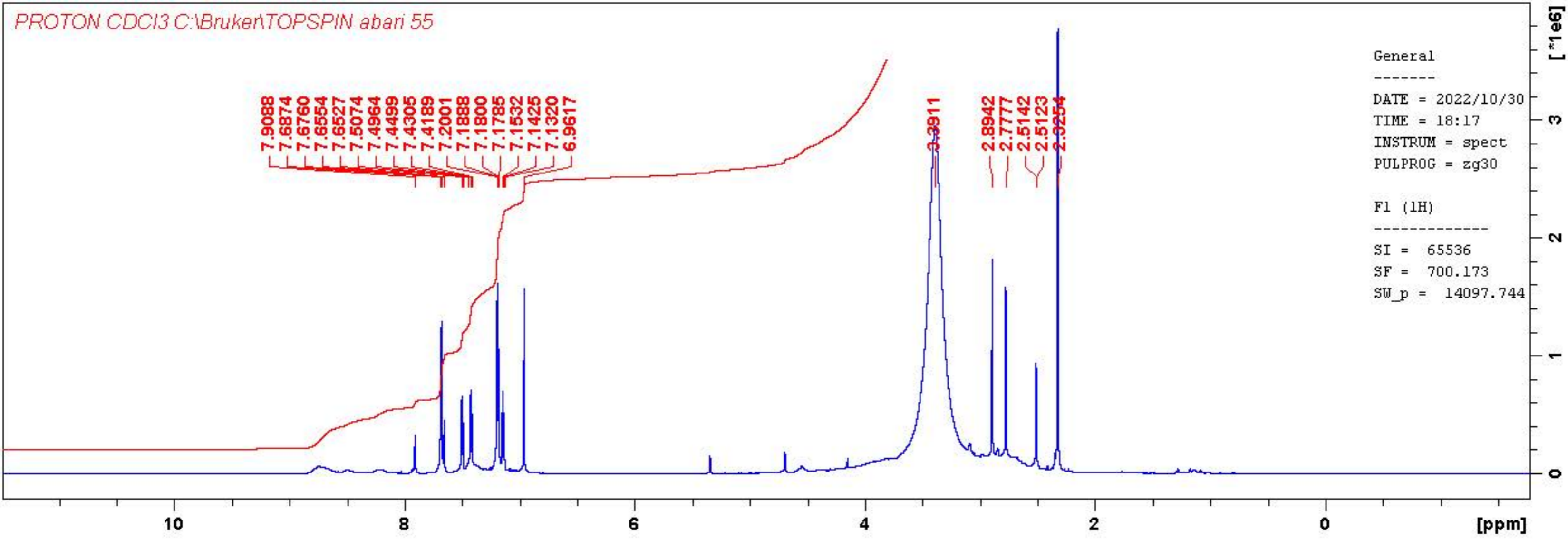

8.1780  
8.1613  
8.0376  
7.9688  
7.9521  
7.7186  
7.7019  
7.6769  
7.6620  
7.6259  
7.6184  
7.6107  
7.6017  
7.5827  
7.5787  
7.5705  
7.5543  
7.5042  
7.4892  
7.4736  
7.4535  
7.4387  
7.4237  
7.4101  
7.3954  
7.3808  
7.3551  
7.3462  
7.3341  
7.3319  
7.3176  
7.3035  
7.3012  
7.2872  
7.2777  
7.2629  
7.2486  
7.2208  
7.0840  
6.9921  
6.9509  
6.9359

2.9693  
2.9029  
2.1919

General  
-----  
DATE = 2022/01/16  
TIME = 13:07  
INSTRUM = spect  
PULPROG = zg30  
  
F1 (1H)  
-----  
SI = 32768  
SF = 500.13  
SW\_p = 10330.579

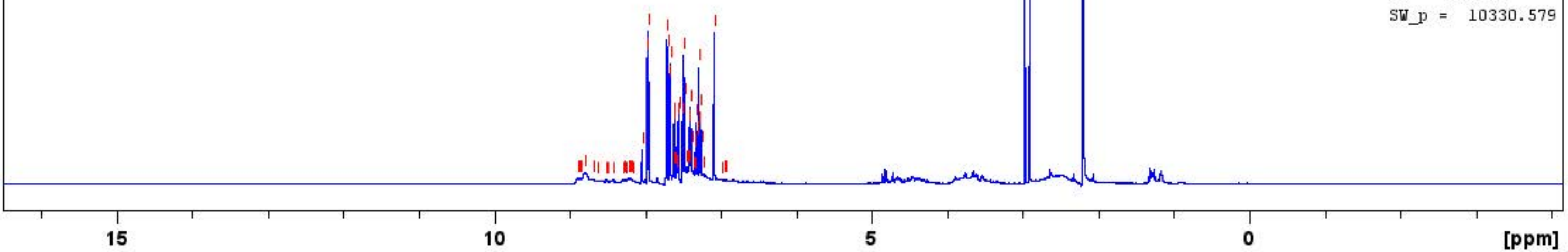

Supplement: S2 File — (PDF) [file pone.0286195.s002.pdf]

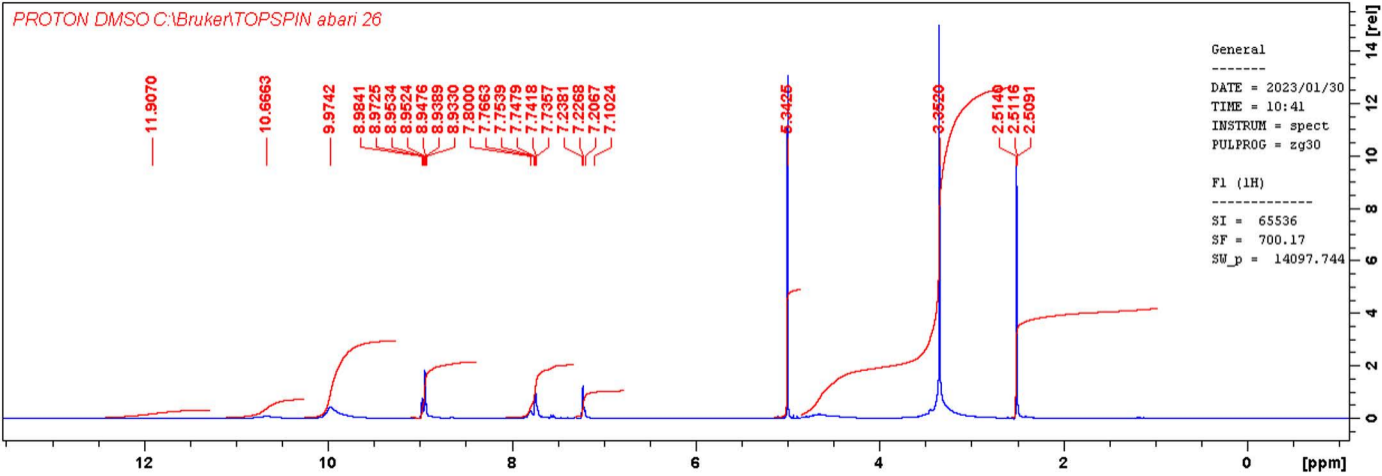

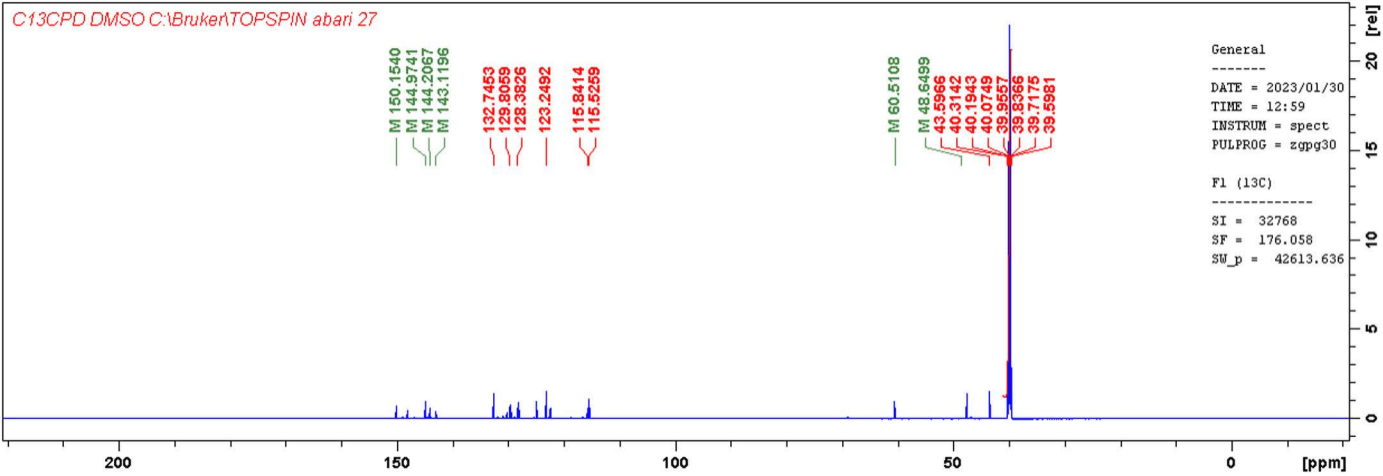

Supplement: S3 File — (PDF) [file pone.0286195.s003.pdf]
